# Foxp3^+^ CD4^+^ regulatory T cells control dendritic cells in inducing antigen-specific immunity to emerging SARS-CoV-2 antigens

**DOI:** 10.1371/journal.ppat.1010085

**Published:** 2021-12-09

**Authors:** Ryuta Uraki, Masaki Imai, Mutsumi Ito, Hiroaki Shime, Mizuyu Odanaka, Moe Okuda, Yoshihiro Kawaoka, Sayuri Yamazaki

**Affiliations:** 1 Department of Immunology, Nagoya City University Graduate School of Medical Sciences, Nagoya, Japan; 2 Division of Virology, Department of Microbiology and Immunology, Institute of Medical Science, University of Tokyo, Minato-ku, Tokyo, Japan; 3 Department of Special Pathogens, International Research Center for Infectious Diseases, Institute of Medical Science, University of Tokyo, Minato-ku, Tokyo, Japan; 4 Department of Pathobiological Science, School of Veterinary Medicine, University of Wisconsin-Madison, Madison, Wisconsin, United States of America; 5 The Research Center for Global Viral Diseases, National Center for Global Health and Medicine Research Institute, Shinjuku-ku, Tokyo, Japan; University of Iowa, UNITED STATES

## Abstract

Regulatory T (Treg) cells, which constitute about 5–10% of CD4^+^T cells expressing Foxp3 transcription factor and CD25(IL-2 receptor α chain), are key regulators in controlling immunological self-tolerance and various immune responses. However, how Treg cells control antigen-specific immunity to severe acute respiratory syndrome coronavirus 2 (SARS-CoV-2) remains unclear. In this study, we examined the effect of transient breakdown of the immunological tolerance induced by Treg-cell depletion on adaptive immune responses against administered SARS-CoV-2 antigen, spike protein 1 (S1). Notably, without the use of adjuvants, transient Treg-cell depletion in mice induced anti-S1 antibodies that neutralized authentic SARS-CoV-2, follicular helper T cell formation and S1-binding germinal center B cell responses, but prevented the onset of developing autoimmune diseases. To further clarify the mechanisms, we investigated maturation of dendritic cells (DCs), which is essential to initiate antigen-specific immunity. We found that the transient Treg-cell depletion resulted in maturation of both migratory and resident DCs in draining lymph nodes that captured S1-antigen. Moreover, we observed S1-specific CD4^+^ T cells and CD8^+^ T cells with interferon-γ production. Thus, captured S1 was successfully presented by DCs, including cross-presentation to CD8^+^ T cells. These data indicate that transient Treg-cell depletion in the absence of adjuvants induces maturation of antigen-presenting DCs and succeeds in generating antigen-specific humoral and cellular immunity against emerging SARS-CoV-2 antigens. Finally, we showed that SARS-CoV-2 antigen-specific immune responses induced by transient Treg-cell depletion in the absence of adjuvants were compatible with those induced with an effective adjuvant, polyriboinosinic:polyribocytidyl acid (poly IC) and that the combination of transient Treg-cell depletion with poly IC induced potent responses. These findings highlight the capacity for manipulating Treg cells to induce protective adaptive immunity to SARS-CoV-2 with activating antigen-presenting DCs, which may improve the efficacy of ongoing vaccine therapies and help enhance responses to emerging SARS-CoV-2 variants.

## Author summary

To challenge SARS-CoV-2 and emerging antigens, it is important to explore innovative approaches to induce effective adaptive immunity to SARS-CoV-2. To induce antigen-specific immunity, vaccines generally need adjuvants to activate dendritic cells (DCs) to present antigens to naïve T cells. In this study, we focused on regulatory T (Treg) cells, which play a key role in maintaining self-tolerance and suppress important immune responses, and found that transient Treg-cell depletion without adjuvants induced protective SARS-CoV-2 antigen-specific immunity. Upon transient Treg-cell depletion in mice, a single administration of SARS-CoV-2 spike protein 1 (S1) induced neutralizing antibodies against live SARS-CoV-2 and evaded the onset of autoimmune diseases. Transient Treg-cell depletion initiated maturation of S1-captured DCs in draining lymph nodes, which is a crucial step to initiate antigen-specific immunity. S1-specific CD4^+^ and CD8^+^T cells that produced interferon-γ were also induced. Furthermore, we showed that transient Treg-cell depletion induced antigen-specific immune responses similar to vaccination with an adjuvant and that the combination induced a heightened effect. Thus, transient breakdown of the immunological tolerance induced by Treg-cell manipulation stimulates an adaptive response by activating DCs, providing an innovative approach to the design of vaccines for SARS-CoV-2 and emerging variants.

## Introduction

The ongoing global pandemic occurred by the coronavirus disease 2019 (COVID-19) whose causative agent is severe acute respiratory syndrome coronavirus 2 (SARS-CoV-2) has led to a worldwide public health and economic damages, and SARS-CoV-2 continues to spread throughout the world. Since outcomes of COVID-19 patients and responses to vaccines are dependent on antigen-specific immunity [1–6], it is crucial to understand how antigen-specific immunity against SARS-CoV-2 is controlled.

Regulatory T (Treg) cells, which express the transcription factor Foxp3 and CD25(IL-2 receptor α chain), constitute 5–10% of CD4^+^T cells and maintain immunological self-tolerance [7–11]. Treg cells not only suppress autoimmune diseases, but also play important roles in regulating various immune responses, inflammation and tissue homeostasis [11–14]. However, it is still to be determined how Treg cells influence on antigen-specific immune responses against SARS-CoV-2. We and others showed that manipulating Treg function by blockade or depletion induces autoimmunity as well as effective anti-tumor immunity in mice [15–19]. Therefore, the breakdown of Treg cell-mediated tolerance may be able to trigger antigen-specific immunity to emerging antigens such as SARS-CoV-2.

In the present study, we aimed to examine the role of Treg cells in inducing antigen-specific immunity against SARS-CoV-2 using Treg cell-sufficient or transient Treg cell-depleted mice with administration of SARS-CoV-2 antigens. We investigated humoral and cellular immune responses as well as dendritic cell (DC), which is an essential antigen-presenting cell to induce antigen-specific immunity [20–24] and also maintain Treg cells [25–27]. Exploring the underlying mechanisms that control antigen-specific immunity should contribute to the development of an innovative strategy against emerging antigens, including SARS-CoV-2 and its variants.

## Results

### Transient depletion of Treg cells, without adjuvants, induces antibodies that neutralize authentic SARS-CoV-2

**Fig 1:**
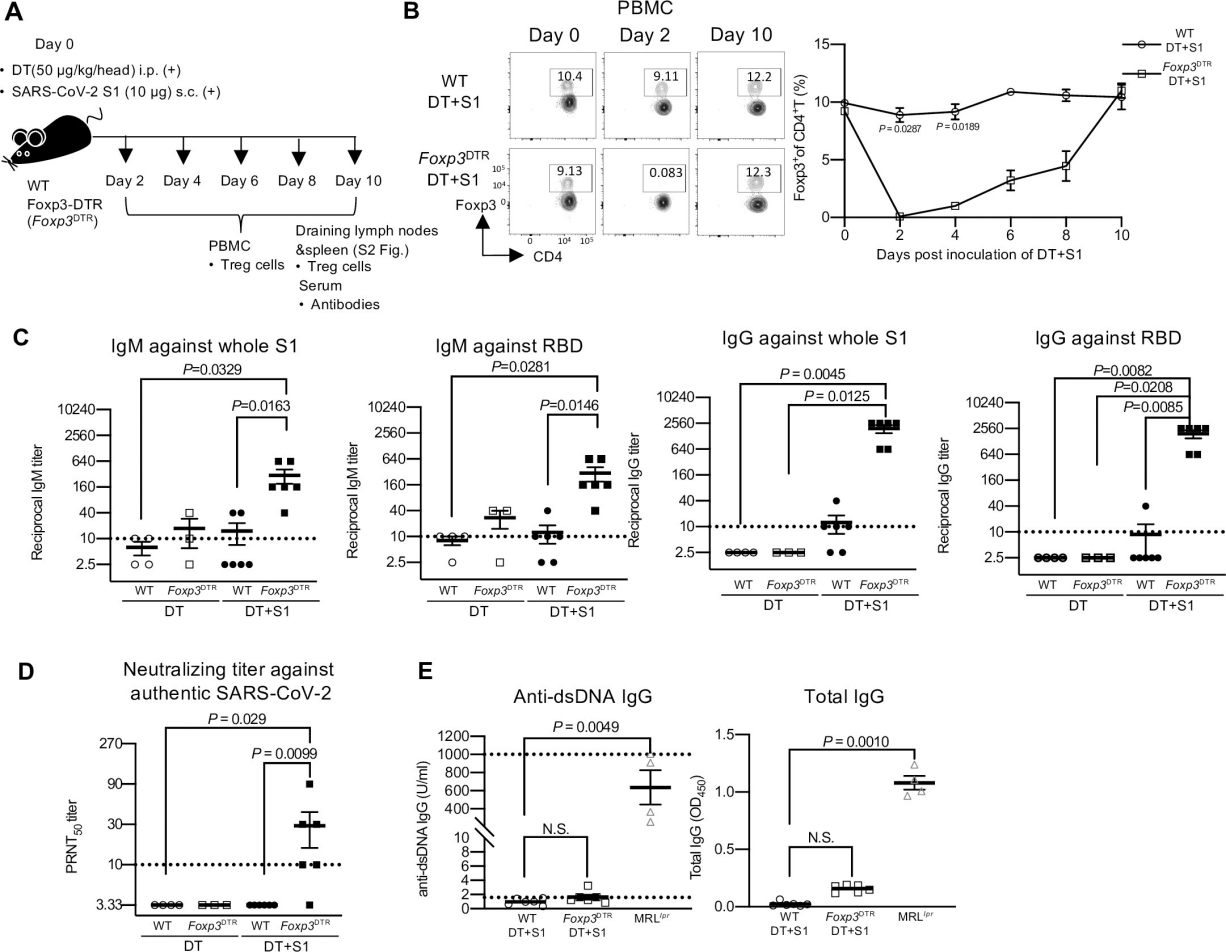
Transient Treg-cell depletion without adjuvants induces neutralizing antibodies to authentic SARS-CoV-2. (A) A schematic diagram showing the experimental workflow. Wild type (WT) and Foxp3-DTR (*Foxp3*^DTR^) mice were injected with diphtheria toxin (DT) (50 μg/kg) intraperitoneally (i.p.) and SARS-CoV-2 spike protein 1 (S1) (10 μg) subcutaneously (s.c.) into four footpads on day 0. At the indicated days, peripheral blood mononuclear cells (PBMCs), draining lymph nodes (axillary and popliteal), spleen and sera were analyzed. (B) PBMCs were analyzed for Foxp3 at the indicated time points. Representative FACS plots from two independent experiments, gated on CD45^+^ CD4^+^ cells, are shown (S1A Fig, for gating strategy). Representative cell frequency data from two separate experiments are shown (n = 3/group). Data are presented as the mean ± SEM, two-way ANOVA followed by Bonferroni’s multiple comparisons test. (C) The levels of whole S1 or receptor-binding domain (RBD)-specific IgM and IgG in sera on day 10 post-injection were determined by ELISA. Viral antibody endpoint titers against S1 and RBD were expressed as the reciprocal of the highest dilution with an optical density at 490 nm (OD490) cutoff value > 0.1. Sera obtained from three independent experiments were analyzed. Data represents the mean ± SEM (DT -injected WT mice, n = 4; DT -injected *Foxp3*^DTR^ mice, n = 3; DT+S1-injected WT mice, n = 6; DT+S1-injected *Foxp3*^DTR^ mice, n = 6). Data were analyzed using the Kruskal-Wallis test with Dunn’s multiple comparisons test. (D) Levels of neutralizing antibodies to authentic SARS-CoV-2 in sera on day 10 post-injection were analyzed by plaque reduction neutralization tests (PRNT). The highest dilution reducing plaque numbers by 50% (PRNT_50_) titers were determined. Data represent the mean ± SEM from three independent experiments (DT-injected WT mice, n = 4; DT-injected *Foxp3*^DTR^ mice, n = 3; DT+S1-injected WT mice, n = 6; DT+S1-injected *Foxp3*^DTR^ mice, n = 6). Data were analyzed using the Kruskal-Wallis test with Dunn’s multiple comparisons test. The horizontal broken line indicates the detection limit. (E) Levels of anti-dsDNA IgG and total IgG in sera on day 10 post-injection were measured by ELISA. Data represent the mean ± SEM from three independent experiments (DT+S1-injected WT mice, n = 6; DT+S1-injected *Foxp3*^DTR^ mice, n = 6). The sera from 10-week female MRL/lpr (MRL^lpr^) mice were used as a positive control (n = 4). The horizontal broken lines in the right panel indicate the detection limits. Kruskal–Wallis test with Dunn’s multiple comparisons test.

To examine the effect of transient breakdown of Treg tolerance on the induction of SARS-CoV-2 antigen-specific immunity, we used Foxp3-IRES-DTR-GFP (*Foxp3*^DTR^) mice in which Foxp3^+^ Treg cells can be depleted by injection of diphtheria toxin (DT) [28]. *Foxp3*^DTR^ mice and wild-type (WT) mice were injected intraperitoneally (i.p.) with DT and subcutaneously (s.c.) with full-length spike protein 1 of SARS-CoV-2 (S1) without adjuvants on day 0 (Fig 1A). We confirmed that Foxp3^+^ CD4^+^ Treg cells in *Foxp3*^DTR^ mice were depleted at 2 days and recovered at day 10 post injection (Figs 1B, S1A for gating strategy, and S2.).

Next, we examined whether anti-S1-specific antibodies were elicited in transient Treg cell-depleted mice (DT+S1-injected *Foxp3*^DTR^ mice) at 10 days post-injection. We measured the IgG and IgM responses against the purified S1 and the receptor-binding domain (RBD) of the S1 protein, which is the main target of neutralizing antibodies to the SARS-CoV-2 [1,29–32]. Surprisingly, DT+S1-injected *Foxp3*^DTR^ mice elicited substantial amounts of S1- and RBD-reactive IgM and IgG compared to Treg-sufficient mice (DT+S1-injected WT mice) (Fig 1C). There was no significant difference in the S1- and RBD-reactive IgM production between DT-only-injected WT and *Foxp3*^DTR^ mice (Fig 1C). To further investigate the quality and function of the induced antibodies in DT+S1-injected *Foxp3*^DTR^ mice, we took advantage of a plaque reduction neutralization test (PRNT) against authentic SARS-CoV-2. Notably, most sera from DT+S1-injected *Foxp3*^DTR^ mice (5 out of 6 mice) actively neutralized authentic SARS-CoV-2, while no sera from DT+S1-injected WT mice (0 out of 6 mice) showed neutralizing activity (Fig 1D). Thus, transient Treg-cell depletion induces SARS-CoV-2 antigen-specific antibodies without adjuvants.

We also examined anti-double stranded DNA (dsDNA) antibody as a representative autoantibody, and total IgG levels, because prolonged Treg-cell depletion induces severe autoimmune diseases [28]. Transient Treg-cell depletion did not increase the production of anti-dsDNA IgG and total IgG as compared to the increased production in age-matched MRL/lpr lupus-prone autoimmune mice as positive controls (Fig 1E).

These data indicate that transient Treg-cell depletion succeeds in producing SARS-CoV-2 antigen-specific protective antibody responses without developing autoimmunity.

### Transient depletion of Treg cells produces follicular helper T cell formation and SARS-CoV-2 antigen-specific B cells

**Fig 2:**
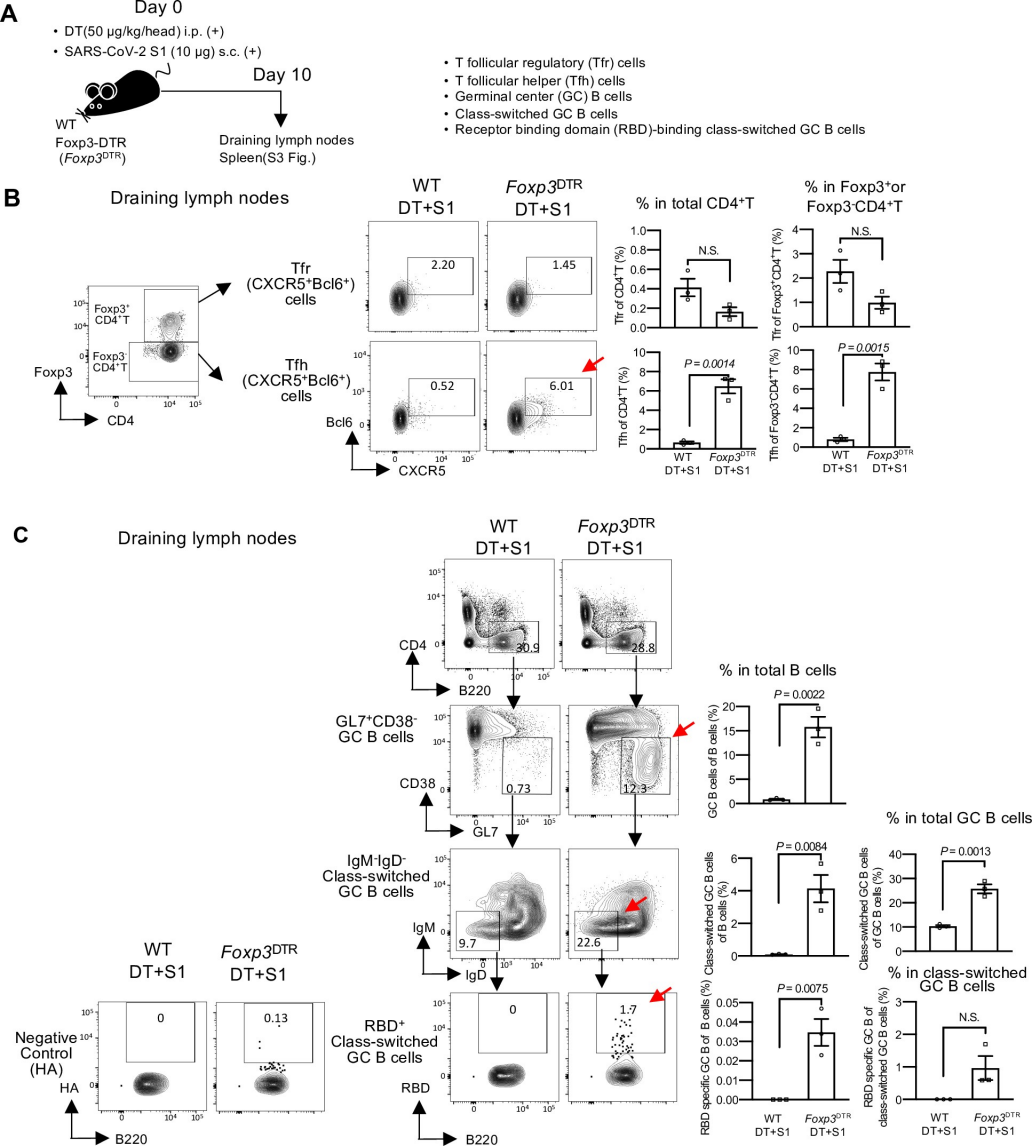
Transient Treg-cell depletion induces the formation of follicular helper T cells and antigen-specific B cells in S1-injected mice. (A) A schematic diagram showing the experimental workflow. As in Fig 1A, but follicular helper T(Tfh), follicular regulatory T (Tfr) and B cells from draining (axillary and popliteal) lymph nodes and spleens were analyzed on day 10 post-injection. (B) Representative gating strategy used to identify Tfr and Tfh cells (S1B Fig, gating strategy). Representative FACS plots of two independent experiments are shown. Representative graphs of two independent experiments are shown as the mean ± SEM (n = 3/group). Data were analyzed using unpaired Student’s t-test. (C) Representative FACS plots of GL7^+^CD38^-^ germinal center (GC) B cells gated on live B220^+^ CD4^-^ cells (S1C Fig, gating strategy). IgM^-^ IgD^-^ class-switched GC B cells were gated on GL7^+^ CD38^-^ GC B cells. RBD-binding class-switched GC B cells were gated on IgM^-^ IgD^-^ class-switched GC B cells. Influenza virus HA protein was used as the negative control for RBD-binding. Representative FACS plots of two independent experiments are shown. Representative frequencies of GC B, class-switched GC B, and RBD-binding GC B cells from two independent experiments are plotted as the mean ± SEM (n = 3/group). Data were analyzed using unpaired Student’s t-test.

As it was of note that Treg-cell depletion without adjuvants induced neutralizing antibodies to administered S1, we examined follicular helper T (Tfh) and B cells (Fig 2A). Tfh cells, defined by CXCR5 and Bcl6 expression in Foxp3^-^ CD4^+^, are the major helper T cells that induce the humoral immune response by producing antigen-specific B cells [33,34]. T follicular regulatory (Tfr) cells are a subset of Foxp3^+^Treg cells expressing CXCR5 and Bcl6, and control Tfh cells [35–37]. In DT+S1-injected *Foxp3*^DTR^ mice on day 10, the formation of Tfh cells was enhanced in both draining lymph nodes and spleen (red arrows in Figs 2B, S3A and S1B for gating strategy), whereas the frequency of Tfr cells was not significantly changed (Fig 2B, S3A and S1B for gating strategy). We also investigated germinal centers (GCs), where high-affinity antibody-producing cells and class-switched GC B cells develop [33,34]. In both draining lymph nodes and spleens on day 10, the frequencies of B cells in GCs, defined by CD38^-^ GL7^+^ [37], and IgD^-^ IgM^-^class-switched GC B cells were remarkably increased in DT+S1-injected *Foxp3*^DTR^ mice compared to that in DT+S1-injected WT mice (red arrows in Figs 2C, S3B and S1C for gating strategy). To determine whether the class-switched GC B cells could recognize RBD, their binding capacity for biotinylated-RBD protein was examined. Notably, in draining lymph nodes, DT+S1-injected *Foxp3*^DTR^ mice produced significantly higher levels of class-switched GC B cells recognizing the RBD of S1 protein (red arrow in Fig 2C, bottom), but much lower levels of the influenza virus hemagglutinin (HA) control protein (Figs 2C, bottom left and S4.). The small number of HA-binding B cells in DT+S1-injected *Foxp3*^DTR^ mice was significantly higher than that in DT+S1-injected WT mice (S4 Fig), suggesting that transient Treg-cell depletion may non-specifically stimulate antibody production. These data demonstrate that transient Treg-cell depletion with SARS-CoV-2 S1 administration induces the formation of Tfh and antigen-specific B cell maturation, which are essential for the induction of humoral immunity.

### Transient depletion of Treg cells induces DC maturation without adjuvants

**Fig 3:**
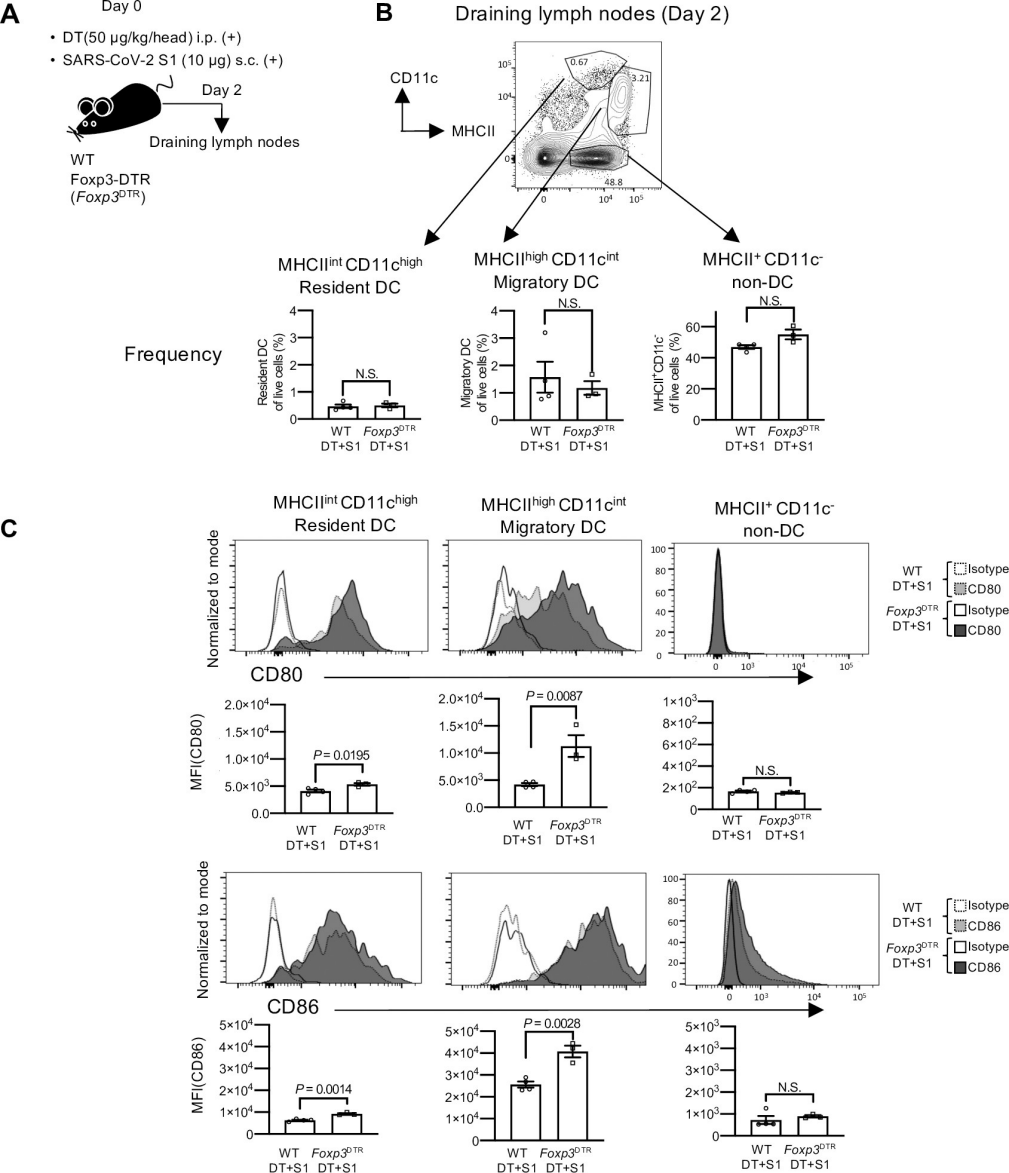
DCs undergo maturation without adjuvants in transient Treg cell-depleted mice. (A) A schematic diagram showing the experimental workflow. As in Fig 1A, but dendritic cells (DCs) from draining lymph nodes (axillary and popliteal) were analyzed on day 2. (B) A representative FACS plot from two independent experiments is shown for MHCII^high^ CD11c^int^ migratory DCs, MHCII^int^ CD11c^high^ resident DCs, and MHCII^+^ CD11c^-^ non-DCs gated on CD45^+^ cells (S1D Fig, gating strategy). Frequency of resident DCs, migratory DCs and MHCII^+^ CD11c^-^ non-DCs from draining lymph nodes of DT+S1-injected WT and *Foxp3*^DTR^ mice. The data are represented summaries from two independent experiments as the mean ± SEM (DT+S1-injected WT mice, n = 4; DT+S1-injected *Foxp3*^DTR^ mice, n = 3). Data were analyzed using unpaired Student’s t-test. (C) Representative histograms from two independent experiments measuring CD80 (top) and CD86 (bottom) in resident DCs, migratory DCs, and non-DCs are shown. The graphic data showing the mean fluorescence intensity (MFI) are summarized from two independent experiments (DT+S1-injected WT mice, n = 4; DT+S1-injected *Foxp3*^DTR^ mice, n = 3). Data were analyzed using unpaired Student’s t-test.

To investigate the mechanisms that induce antigen-specific immunity by transient Treg-cell depletion, DCs were investigated. DCs capture exogenous antigens, undergo maturation and present processed antigens to naïve T cells, leading to antigen-specific immunity [20–23]. After 2 days of DT and S1 injection, the frequency and maturation status of DCs in the draining lymph nodes were investigated (Fig 3A). Although the frequencies of migratory DCs, defined as MHC class II (MHCII)^high^ CD11c^intermediate (int)^, and resident DCs, defined as MHCII^int^ CD11c^high^ were similar between DT+S1-injected *Foxp3*^DTR^ and WT mice (Figs 3B and S1D for gating strategy), the expression levels of the main co-stimulatory molecules, CD80 and CD86, were significantly higher in DT+S1-injected *Foxp3*^DTR^ mice, demonstrating that transient Treg-cell depletion without adjuvants successfully induced the maturation of both migratory and resident DCs (Figs 3C and S1D for gating strategy). The expression levels of both CD80 and CD86 in MHCII^+^ CD11c^-^ non-DCs, mostly macrophages and B cells, were much lower than those in DCs (Fig 3C, right). These data demonstrate that the drastic increase in CD80 and CD86 expression was specific to DCs in transient Treg-depleted mice.

Aside from migratory or resident DCs, DCs can be divided into several subsets with prominent functions, distinguished by surface markers such as CD11b^+^ cDC2 or CD8^+^ cDC1 subsets [21,23,24]. There were no significant changes in their frequency (S5A Fig), but CD80 and CD86 expression was similarly upregulated in both CD11b^+^ and CD8^+^ subsets in migratory and resident DCs in DT+S1-injected *Foxp3*^DTR^ mice compared to that in DT+S1-injected WT mice (S5B Fig).

On the other hand, interestingly, partial Treg-cell depletion using an anti-CD25 monoclonal antibody (mAb) [15] (S6B Fig), which reduced the frequency of Treg cells by 50% at 2–10 days post antibody injection (S5B Fig), neither induced DC maturation nor enhanced the production of S1- and RBD-reactive antibodies (S6C and S6D Fig).

Thus, transient, but complete, Treg-cell depletion is sufficient to initiate maturation of DCs and induces functional antibodies to administered S1 antigens.

### Transient depletion of Treg cells induces the maturation of SARS-CoV-2 protein-captured DCs without adjuvants

**Fig 4:**
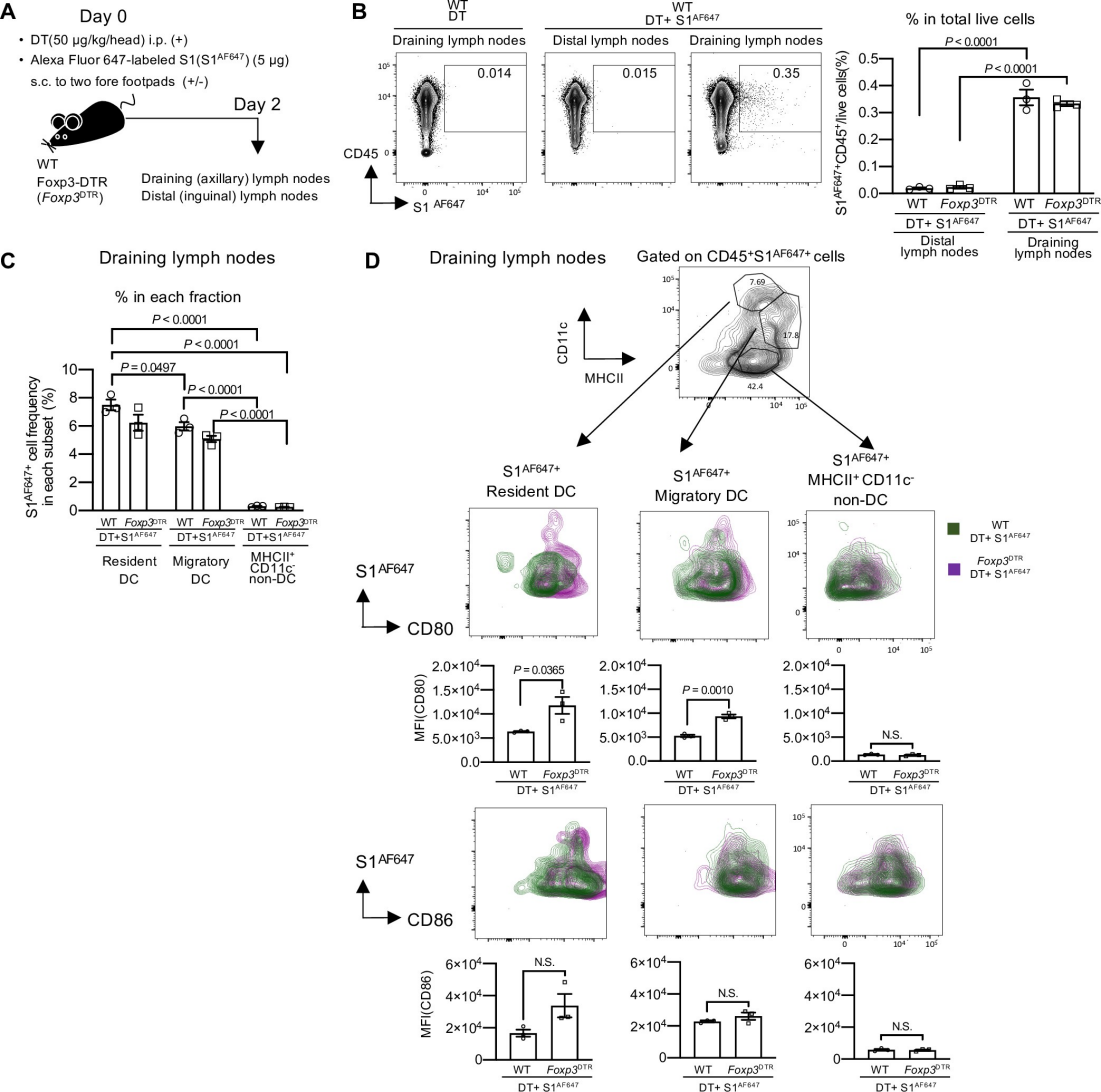
DCs capture S1 protein and undergo maturation in transient Treg cell-depleted mice. (A) A schematic diagram showing the experimental workflow. WT and *Foxp3*^DTR^ mice were injected with DT (50 μg/kg) i.p. and Alexa Fluor 647 labeled S1(S1^AF467^) (5 μg) s.c. into two fore footpads on day 0. Cells from draining (axillary) lymph nodes and distal (inguinal) lymph nodes were analyzed on day 2. (B) Representative FACS plots of S1^AF647+^ cells in the distal and draining lymph nodes from WT mice on day 2. Cells were gated on live cells. Percentage of S1^AF647+^ CD45^+^ cells / live cells in distal and draining lymph nodes from DT+S1 ^AF647^-injected WT and *Foxp3*^DTR^ mice. Representative data from two independent experiments were plotted as the mean ± SEM (n = 3 mice /group). Data were analyzed using two-way ANOVA with Tukey’s multiple comparisons test. (C) Percentage of S1^AF647+^ cells in resident DCs, migratory DCs or MHCII^+^ CD11c^-^ non-DCs in draining lymph nodes from DT+S1 ^AF647^-injected WT and *Foxp3*^DTR^ mice. Representative data from two independent experiments is plotted as the mean ± SEM (n = 3 mice /group). Data were analyzed using two-way ANOVA with Tukey’s multiple comparisons test. (D) A representative FACS plot to identify migratory DCs, resident DCs, and MHCII^+^ CD11c^-^ non-DCs, gated on live CD45^+^ S1^AF647+^ cells from draining lymph nodes of DT+S1^AF647^-injected *Foxp3*^DTR^ mice are shown (top). Representative overlaid contour plots of CD80 and CD86 expression gated on S1^AF647+^ resident DCs, S1^AF647+^ migratory DCs or S1^AF647+^ MHCII^+^ CD11c^-^ non-DCs from draining lymph nodes of DT+S1 ^AF647^-injected WT or *Foxp3*^DTR^ mice are shown (WT, green; *Foxp3*^DTR^, red-purple). Representative of two independent experiments for FACS plots and graphics are shown. The graphic data are presented as the mean ± SEM of the MFI (n = 3 / group). Data were analyzed using unpaired Student’s t-test.

To induce antigen-specific immunity, it is essential for DCs to capture antigens and present them to naïve T cells [20–23]. To examine whether administered S1 proteins were captured by DCs in transient Treg cell-depleted mice successfully, on the day of DT injection, mice were s.c. injected into both fore footpads with SARS-CoV-2 S1 protein labeled with a fluorescent dye, Alexa Fluor 647 (S1^AF647^) (Fig 4A). We detected S1^AF647+^ CD45^+^ leukocyte cells in draining (axillary) lymph nodes, but not in the distal (inguinal) lymph nodes at day 2 in both DT+S1^AF647^-injected *Foxp3*^DTR^ and WT mice (Fig 4B). Within CD45^+^ leukocyte cells, S1^AF647^ was preferentially captured by MHCII^+^ CD11c^+^ DCs, both migratory and resident DCs, compared to MHCII^+^ CD11c^-^ non-DCs (Fig 4C). In addition, the frequencies of S1^AF647+^ in migratory and resident DCs were similar between DT+S1^AF647^-injected *Foxp3*^DTR^ and WT mice (Fig 4C). However, we found that the expression levels of CD80 in both S1^AF647^-captured migratory and resident DCs were remarkably higher in DT+S1^AF647^-injected *Foxp3*^DTR^ mice than in DT+S1^AF647^-injected WT mice (Fig 4D, middle). CD86 expression levels also tended to be higher in S1^AF647^-captured migratory and resident DC subsets in DT+S1^AF647^-injected *Foxp3*^DTR^ mice, although the difference was not significant (Fig 4D, bottom). These results suggest that transient Treg-cell depletion induced maturation of S1-captured DCs, suggesting effective antigen presentation.

We further investigated the maturation of S1^AF647^-captured CD8^+^ or CD11b^+^ subsets within migratory and resident DCs. Although there was no significant difference in the frequency of S1^AFF647+^ cells in the CD11b^+^ and CD8^+^ DC subsets (S7A and S7B Fig), S1^AF647^-captured CD8^+^ resident DCs and CD11b^+^ migratory DCs in draining lymph nodes had significantly higher expression of CD80 with transient Treg-cell depletion (S7C Fig, top). There was no significant difference in CD86 expression (S7C Fig, bottom). These results suggest that mature CD8^+^ resident DCs and CD11b^+^ migratory DCs may play important roles in antigen presentation.

### Transient depletion of Treg cells successfully induces functional SARS-CoV-2-specific CD4^+^ and CD8^+^T cells

**Fig 5:**
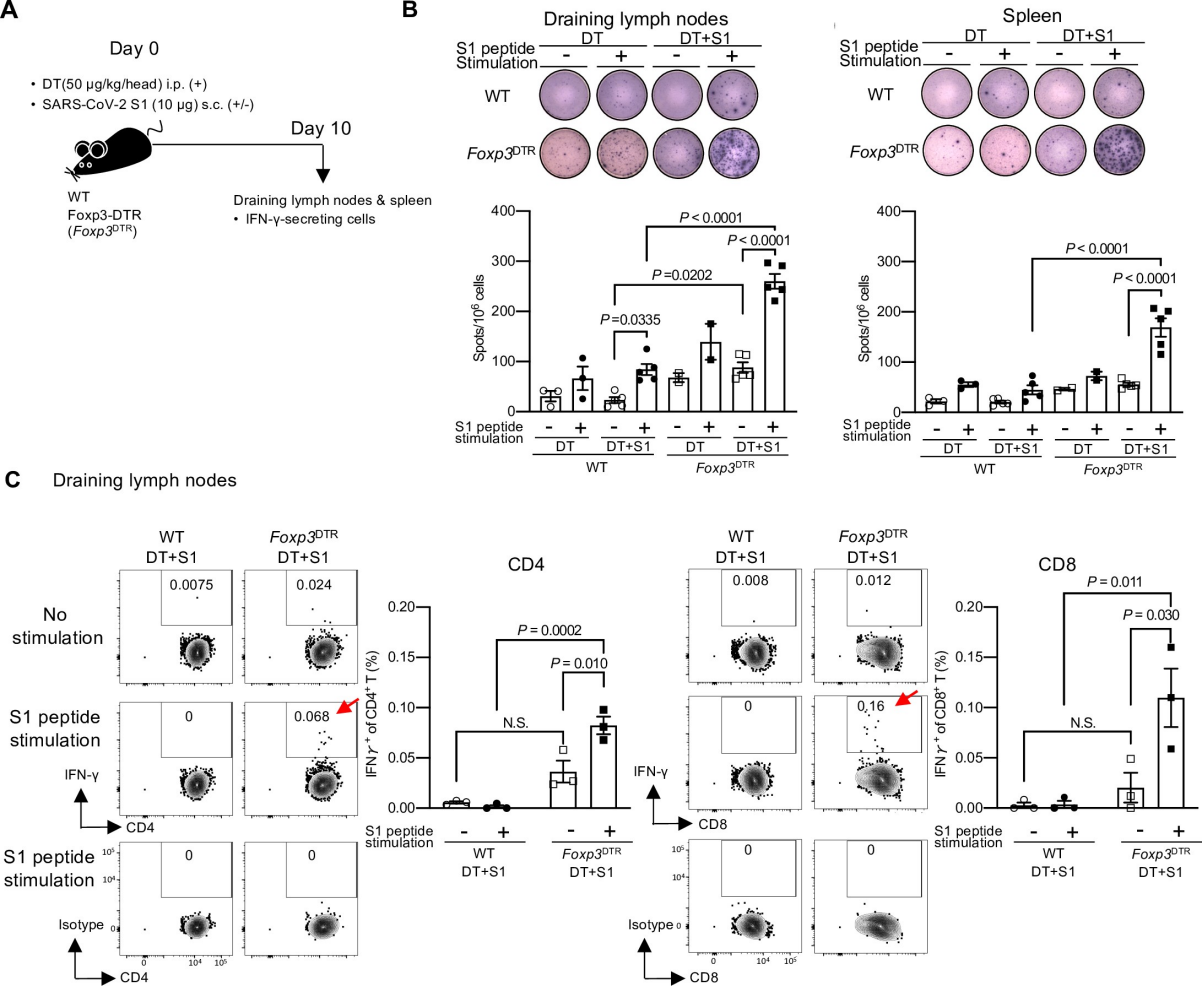
SARS-CoV-2 antigen-specific IFN-γ producing CD4^+^ and CD8^+^ T cells are successfully induced in transient Treg cell-depleted mice. (A) A schematic diagram showing the experimental workflow. As in Fig 1A, but IFN-γ-secreting cells in draining lymph nodes (axillary and popliteal) and spleen on day 10 were analyzed using ELISpot and intracellular cytokine staining. (B) Representative pictures of IFN-γ ELISpot assays from draining lymph nodes and spleens on day 10 following S1 and DT injection are shown (top). The plates of the ELISpot Kit are from R&D Systems Inc.. IFN-γ-secreting spots were quantified after 36 h of stimulation with 1 μg/mL S1 peptide pool. The graphic data are a summary of two independent experiments. The data are represented as the mean ± SEM (DT-injected WT mice, n = 3; DT-injected *Foxp3*^DTR^ mice, n = 2; DT+S1-injected WT mice, n = 5; DT+S1-injected *Foxp3*^DTR^ mice, n = 5). Data were analyzed using two-way ANOVA with Tukey’s multiple comparisons test. (C) IFN-γ-secreting cells from draining lymph nodes on day 10 were analyzed by flow cytometry after 6 h of stimulation with 1 μg/mL S1 peptide pool. Representative FACS plots from two independent experiments are shown for CD4^+^ (left) and CD8^+^ T (right) cells, gated on live CD45^+^, CD3^+^ cells (S1E Fig, gating strategy). The graphic data of the frequencies of IFN-γ positive cells are representative from two independent experiments (n = 3/group). The mean ± SEM is shown. Data were analyzed using two-way ANOVA with Tukey’s multiple comparisons test.

As transient Treg-cell depletion induces S1-captured DC maturation, we examined the S1-specific cell-mediated immune response (Fig 5A). We first measured the numbers of S1-specific interferon γ (IFN-γ)-producing spots in draining lymph nodes and spleens at 10 days post S1 injection in mice treated with DT by enzyme-linked immunospot (ELISpot). S1-specific IFN-γ-producing spots, detected by S1 peptide stimulation, were higher in both organs from DT+S1-injected *Foxp3*^DTR^ mice compared to those from DT+S1-injected WT mice (Fig 5B). In draining lymph node cells, even without S1 peptide stimulation, the frequency of IFN-γ-producing cells was significantly higher in DT+S1-injected *Foxp3*^DTR^ mice than in DT+S1-injected WT mice (Fig 5B).

Next, to confirm whether the IFN-γ-producing cells in draining lymph nodes and spleens were T cells, intracellular cytokine staining by flow cytometry was performed. Notably, with S1 peptide stimulation, DT+S1-injected *Foxp3*^DTR^ mice induced significantly higher frequencies of SARS-CoV-2 S1-specific IFN-γ-producing CD4^+^ and CD8^+^ T cells (red arrows in Figs 5C, S8 and S1E for gating strategy). We also investigated IFN-γ production by CD3^-^ cells, which include natural killer (NK) cells. In DT+S1-injected *Foxp3*^DTR^ mice, more CD3^-^ cells, probably NK cells, produced IFN-γ without S1 peptide stimulation (S9 and S1E Figs for gating strategy).

Thus, transient Treg-cell depletion resulted in antigen non-specific killing activity including NK cells, but also induced the significant expansion of SARS-CoV-2 antigen-specific CD4^+^ and CD8^+^ T cells with IFN-γ production, indicating successful cross-presentation of S1 antigens to CD8^+^T cells by DCs.

### Transient Treg-cell depletion induces a comparable effect with poly IC in inducing SARS-CoV-2 antigen-specific immunity, and the combination shows enhanced effects

**Fig 6:**
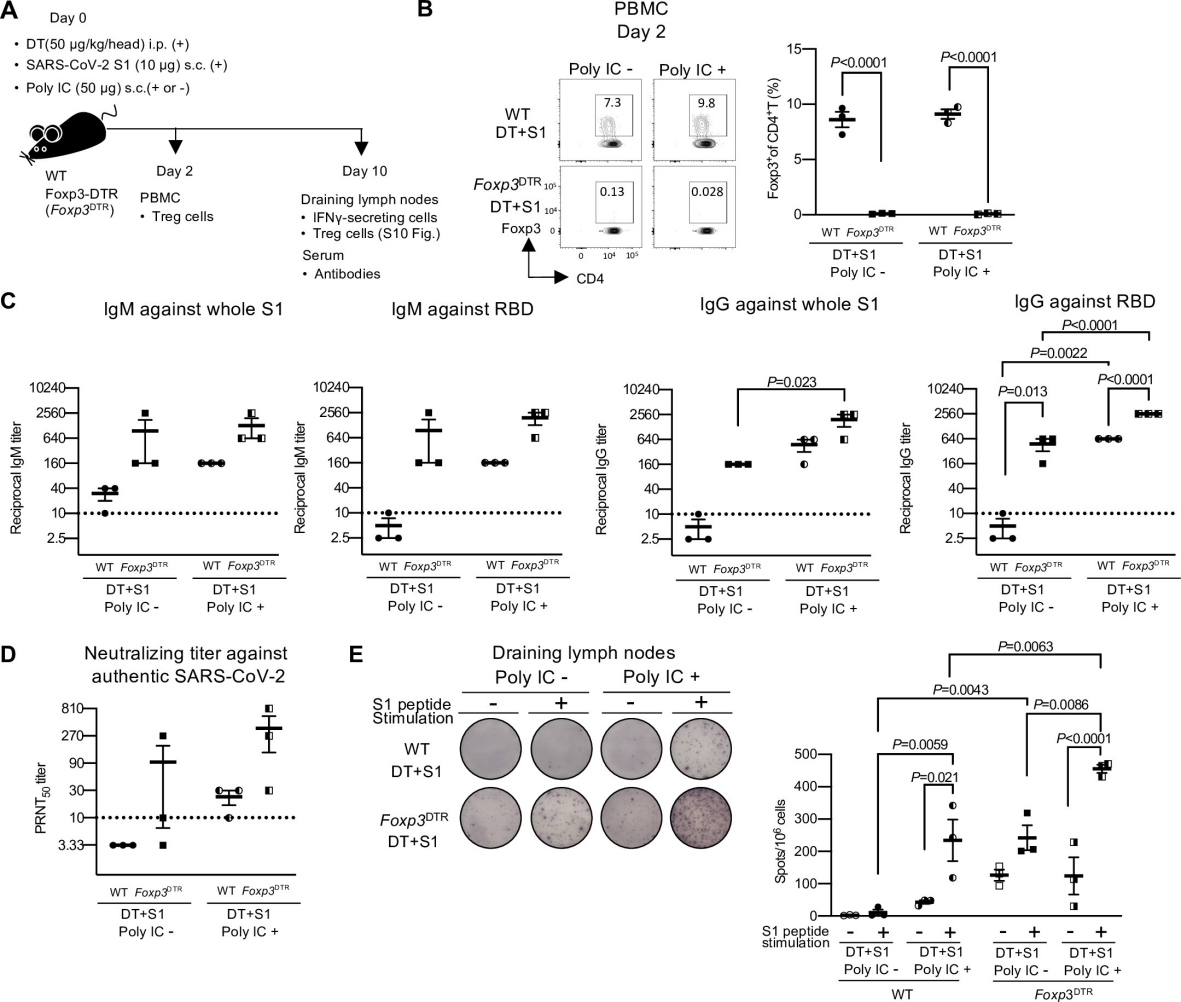
Transient Treg-cell depletion induces humoral and cellular immune responses to SARS-CoV-2 similar to poly IC, and the combination of both can enhance the response. (A) A schematic diagram showing the experimental workflow. As in Fig 1A, but WT and Foxp3^DTR^ mice were injected with DT i.p. and S1 s.c. with or without poly IC (50 μg) s.c. into four footpads on day 0. PBMCs were analyzed for Treg-cell depletion on day 2. On day 10, sera and draining lymph nodes (axillary and popliteal) were analyzed for antibodies and IFN-γ-secreting cells, respectively. (B) PBMCs were analyzed for Foxp3 on day 2. Representative FACS plots from a single independent experiment, gated on CD45^+^ CD4^+^ cells, are shown (S1A Fig for gating strategy). Data are presented as mean ± SEM (n = 3 /group). Data were analyzed using two-way ANOVA followed by Tukey’s multiple comparisons test. (C) Levels of whole S1 or RBD-specific IgM and IgG in sera on day 10 post-injection were determined by ELISA. Viral antibody endpoint titers against S1 and RBD were expressed as the reciprocal of the highest dilution with an optical density at 490 nm (OD_490_) cutoff value > 0.1. Sera obtained from a single experiment were analyzed. Data represents the mean ± SEM from a single experiment (n = 3 /group). Data were analyzed using two-way ANOVA followed by Tukey’s multiple comparisons test. The horizontal broken line indicates the detection limit. (D) The levels of neutralizing antibodies to authentic SARS-CoV-2 in sera on day 10 post-injection were analyzed by PRNT. The highest dilution reducing plaque numbers by 50% (PRNT50) titers were determined. Data represent the mean ± SEM from a single experiment (n = 3 /group). Data presented as mean ± SEM. Data were analyzed by two-way ANOVA followed by Tukey’s multiple comparisons test. The horizontal broken line indicates the detection limit. (E) Representative pictures of IFN-γ ELISpot assays from draining lymph nodes and spleens on day 10 following S1 and DT injection with or without poly IC are shown (left). The plates of the ELISpot Kit are from Abcam Inc.. IFN-γ-secreting spots were quantified after 36 h of stimulation with or without 1 μg/mL S1 peptide pool. The right graphics represent mean ±SEM from a single experiment (n = 3 /group). Data were analyzed using two-way ANOVA with Tukey’s multiple comparison.

Finally, we compared the antigen-specific immune responses to S1 protein induced by transient Treg-cell depletion with vaccination with an adjuvant. We chose a synthetic double-stranded RNA, polyriboinosinic:polyribocytidyl acid (poly IC) as an adjuvant because poly IC is an effective adjuvant for DC-targeted protein vaccines [38,39]. DT+S1-injected WT or *Foxp3*^DTR^ mice were injected with or without poly IC s.c. and the antibodies against S1 protein and IFN-γ producing cells were investigated (Fig 6A). We demonstrated transient Treg-cell depletion by peripheral blood mononuclear cells (PBMCs) on day 2 (Fig 6B) and lymph nodes on day 10 (S10 Fig). Treg cells recovered on day 10 in the draining lymph nodes were significantly higher with poly IC (S10 Fig), suggesting that the Treg cells increased to suppress the inflammation [10]. Notably, the amounts of S1- and RBD-reactive IgM and IgG in DT+S1-injected *Foxp3*^DTR^ mice without poly IC (DT+S1-injected *Foxp3*^DTR^) were comparable to those from DT+S1-injected WT mice with an effective adjuvant, poly IC (DT+S1+poly IC-injected WT) (Fig 6C). Furthermore, when transient Treg-cell depletion was combined with poly IC (DT+S1+poly IC-injected *Foxp3*^DTR^ mice), the amounts of S1- and RBD-reactive IgG were notably higher than in DT+S1-injected *Foxp3*^DTR^ mice or DT+S1+poly IC-injected WT mice (Fig 6C, right two graphs). A similar effect was observed for neutralizing activity against live authentic SARS-CoV-2 (Fig 6D). Moreover, in draining lymph nodes, SARS-CoV-2 antigen-specific IFN-γ producing cells were also comparable between transient Treg-depleted mice without poly IC (DT+S1-injected *Foxp3*^DTR^) and vaccinated Treg-sufficient mice (DT+S1+poly IC-injected WT) (Fig 6E). SARS-CoV-2 antigen-specific IFN-γ producing cells were significantly higher in DT+S1+poly IC-injected *Foxp3*^DTR^ mice than in DT+S1 -injected *Foxp3*^DTR^ mice or DT+S1+poly IC-injected WT mice (Fig 6E).

These results indicate that transient Treg-cell depletion induces SARS-CoV-2 antigen-specific humoral and cellular immunities similar to vaccination with poly IC and that the combination with transient Treg-cell depletion can enhance the responses of vaccines to SARS-CoV-2.

## Discussion

In the present study, we found that transient Treg-cell depletion with S1 antigen administration in mice successfully induced the maturation of S1-captured DCs at day 2 and S1-specific humoral and cellular immunity at day 10, without the use of adjuvants. Notably, the elicited antibodies in S1-administered transient Treg-depleted mice had binding affinity to the SARS-CoV-2 S1 protein, and neutralizing activity to the authentic SARS-CoV-2 S1 virus. Furthermore, the SARS-CoV-2 antigen-specific immune responses induced by transient Treg-cell depletion with S1 antigen administration were comparable to those induced by the vaccination with an effective adjuvant, poly IC. These results suggest that Treg cells can be targets for promoting SARS-CoV-2 antigen-specific immunity, which may be applied to vaccine development to enhance the efficacy against the original virus as well as emerging variants.

Treg cells maintain immunological self-tolerance and play a key role in controlling the homeostasis of the immune system [10]. Treg cells also contribute to the regulation of the immune response to pathogens, but the roles of Treg cells differ depending on the infectious model and timing [40–43]. How Treg cells play a role in the immune responses to COVID-19 remains unclear, but it has been reported that Treg cells were expanded in COVID-19 patients [44,45]. Treg cells detected by FOXP3 staining in multi-color immunofluorescence imaging were expanded in the lymph nodes and spleens, in contrast to the loss of GCs after 2–3 weeks of COVID-19 development [44]. A recent study reported that Treg cells, whose signature is similar to those expanded in cancer, were expanded in more severe COVID-19 patients [45]. As Treg cells maintain tissue homeostasis by controlling inflammation and tissue repair [12–14], the expansion of Treg cells in COVID-19 patients could reflect an attempt to suppress ongoing inflammation. It is possible that Treg dysfunction or impaired functional Treg differentiation may correlate with the severity of inflammation or cytokine storm in COVID-19 [46]. A study using an established SARS-CoV-2 infected animal model to find the effect of Treg cells on suppressing inflammation or cytokine storm in COVID-19 is therefore warranted [47–50]. In addition, the expanded Treg cells could cause the impairment of DC maturation in COVID-19 patients [51]. Further studies are therefore required to elucidate the role of Treg cells in COVID-19.

In the present study, we showed that the transient release of DCs from Treg tolerance is sufficient to evoke DC maturation with successful induction of adaptive immunity to administered S1 protein. The upregulation of CD80 and CD86 expression levels in both migratory DCs and resident DCs was observed on day 2 after Treg-cell depletion with S1 protein injection, which subsequently generated S1 antigen-specific CD4^+^ and CD8^+^ T cells on day 10. Notably, the induction of IFN-γ producing antigen-specific CD8^+^ T cells indicated that the S1 protein was cross-presented successfully by mature DCs in transient Treg-depleted mice. CD8^+^ cDC1 subsets are specialized to cross-present exogenous antigens to naïve CD8^+^T cells [20,21,24,52,53]. Consistent with this, we found that Treg-cell depletion induced maturation of S1-captured CD8^+^ resident DCs, which probably play a key role in cross-presenting S1 protein to naïve CD8^+^ T cells. Treg cells suppress DC functions directly by downregulating CD86 and CD80[54–56], and also indirectly by suppressing the effector functions of other immune cells, such as cytokine production [10,28,57,58]. Transient Treg-cell depletion also increased IFN-γ production non-specifically from CD3^-^ cells including NK cells, as we showed previously that Treg-cell depletion induced effective anti-tumor immunity by inducing tumor-specific cytotoxic CD8^+^ T cells and IFN-γ-producing CD4^-^ CD8^-^ cells, including NK cells [15]. Therefore, our data indicate that transient Treg-cell depletion induces SARS-CoV-2 antigen-specific adaptive immunity, together with potentiating the innate immune response, which plays a role in inducing adaptive immunity [20,22]. Further studies are required to investigate the mechanism and direct impact of DC maturation in transient Treg cell-depleted hosts.

Notably, we found that S1 protein administration alone during transient Treg-cell depletion induced functional anti-S1 antibodies with the capacity to neutralize authentic SARS-CoV-2 actively. In contrast, the production of anti-S1 antibodies was not induced by anti-CD25 mAb treatment, although Treg reduction induced by anti-CD25 mAb treatment has the potential to induce effective tumor immunity [15,16]. Effective antibody production may require additional maturation of DCs, or anti-CD25 mAb treatment may affect the availability of IL-2 for other helper T cells, which is required for antibody production [43,59]. In addition, anti-CD25 mAb treatment only depleted CD25^+^ Foxp3^+^ Treg cells, whereas both CD25^+^ and CD25^-^ Foxp3^+^ Treg cells were depleted in Foxp3^DTR^ mice. The remaining CD25^-^ Foxp3^+^ Treg cells may be sufficient to suppress the production of anti-S1 antibodies, because CD25^-^ Foxp3^+^ Treg cells can convert into CD25^+^ Foxp3^+^ Treg cells similarly to CD25^-^ Foxp3^+^ Tfr cells in GCs [60]. Complete GC loss and Treg expansion have been reported in the spleens and lymph nodes of patients who have died of COVID-19, although the mechanisms are still unknown [44]. If the expanded Treg cells in COVID-19 could play a role in suppressing GC formation, a strategy to deplete Treg cells, as shown in the present study, may recover GC formation.

Our results suggest that transient Treg-cell depletion may be useful in combination with existing vaccines, although further studies on long term or memory responses should be conducted. It has been reported that vaccines using peptides or DCs may induce antigen-specific Treg cells, which attenuate the responses [61,62]. Thus, transient Treg-cell depletion may improve the effectiveness of existing vaccines. In humans, anti-CTLA-4, GITR, CCR4 antibodies or small molecules targeting Treg cells might be useful to deplete or block the function of Treg cells [16,17,63–68]. CTLA-4 is constitutively expressed on the surface of Treg cells and plays a key role in their suppressive function [67,69,70]. The anti-CTLA4 mAb, ipilimumab, the first immune checkpoint inhibitor for immunotherapy of cancer patients, blocks negative signals on effector T cells [71], but can also deplete Treg cells or block Treg suppression [69,70]. However, ipilimumab or anti-CCR4 Abs can induce autoimmune disease in cancer patients, and management to treat the adverse effects is therefore required [65,71]. Although further fundamental research and special caution to prevent the development of autoimmune diseases by manipulating Treg cells is required, these strategies might be combined with vaccines.

Overall, our results suggest that transient breakdown of Treg tolerance could be useful to initiate adaptive immunity against emerging antigens, SARS-CoV-2. This strategy may also be an option for individuals who are allergic to adjuvants. Transient Treg-cell depletion might be combined with vaccine therapy, including current ongoing mRNA vaccines or adenovirus-based vaccines, to activate DCs and potentiate SARS-CoV-2 antigen-specific immunity. This combination treatment may enable a reduction in the amount of antigens in vaccines, which would increase the available supply or help respond to emerging SARS-CoV-2 variants.

## Methods

### Ethics statement

All experiments using mice were approved by the Institutional Animal Care and Use Committee of Nagoya City University (approval number: H29M-01). All experiments using SARS-CoV-2 were performed in enhanced biosafety level 3 (BSL3) containment laboratories at the University of Tokyo, which are approved for use by the Ministry of Agriculture, Forestry, and Fisheries, Japan.

### Reagents

Diphtheria toxin (DT), penicillin-streptomycin, o-phenylenediamine dihydrochloride (OPD) substrate tablets, gentamicin phorbol-12-myristate-13-acetate (PMA), ionomycin and IgG from rat serum were purchased from Sigma-Aldrich (St. Louis, MO, USA). Collagenase D was obtained from Roche (Basel, Switzerland). Recombinant SARS-CoV-2 whole S1 protein, the receptor binding domain (RBD) of S1 protein and the biotinylated RBD of S1 protein were obtained from Biolegend (San Diego, CA, USA). Biotinylated influenza virus (A/California/04/2009: Ca04) hemagglutinin (HA) protein was purchased from Sino Biological (Beijing, China). PepTivator SARS-CoV-2 Prot_S1 was purchased from Miltenyi Biotec. (Bergisch Gladbach, Germany). Poly IC HMW VacciGrade was obtained from Invivogen (San Diego, CA, USA).

### Cell lines

VeroE6 (ATCC CRL-1586) was maintained in Eagle’s minimal essential media (MEM) containing 10% fetal calf serum (FCS), 100 μg/ml gentamicin sulfate and 2.5 μg/ml amphotericin B. VeroE6/TMPRSS2 (JCRB1819) cells obtained from the National Institutes of Biomedical Innovation, Health and Nutrition, Japan were maintained in Dulbecco’s modified Eagle’s medium (DMEM) containing 10% FCS, 100 μg/ml gentamicin sulfate and 2.5 μg/ml amphotericin B at 37°C under 5% CO_2._

### Virus

The SARS-CoV-2 strain (UT-NCGM02/Human/2020/Tokyo), which was isolated from a COVID-19 patient in Japan, was propagated in VeroE6 cells in Opti-MEM I (Invitrogen) containing 0.3% bovine serum albumin (BSA) and 1 μg of L-1-tosylamide-2-phenylethyl chloromethyl ketone (TPCK) treated-trypsin/mL as described previously [48].

### Mice

C57BL/6J (B6) mice (7–11 weeks of age) and MRL/MpJJmsSlc-lpr/lpr (MRL/lpr) mice (7 weeks of age) were purchased from Japan SLC, Inc. (Shizuoka, Japan) or CLEA Japan, Inc. (Tokyo, Japan). Foxp3-IRES-GFP-DTR (*Foxp3*^DTR^) mice were obtained from the Jackson Laboratory (Bar Harbor, ME, USA) [28]. Mice were maintained at the Nagoya City University Animal Facility and at the Animal Care Room in Deperament of Immunology at Nagoya City University approved by the Institutional Animal Care and Use Committee of Nagoya City University (approval number: H29M-01). All mice were maintained under specific pathogen-free conditions.

All experimental procedures were performed under anesthesia using isoflurane (Wako Pure Chemical industries, Ltd, Osaka, Japan).

### SARS-CoV-2 S1 administration and DT injection

WT or *Foxp3*^DTR^ mice were i.p.injected with DT at 50 μg/kg on day 0 to deplete Foxp3^+^ Treg cells [28]. Immediately after injection with DT on day 0, mice were s.c. injected with 10 μg of S1 protein in 120 μL PBS into four footpads.

### Cell prepataion and flow cytometry

To harvest single cells including DCs, spleen and lymph nodes were incubated with collagenase D [25,72,73]. Cells were then incubated with anti-CD16/32 Ab (93) to block Fc receptors and stained with antibodies specific to CD3 (17A2), CD45 (30-F11), CD4 (RM4-5), CD11b (M1/70), CD8a (53–6.7), CD11c (N418), I-A/I-E (M5/114.15.2), CXCR5 (L138D7), CD80 (16-10A1), CD86 (GL-1) from Biolegend or eBioscience(San Diego, CA, USA) and Live/Dead fixable aqua (Thermo Fisher Scientific, Waltham, MA, USA). Abs specific to Foxp3 (FJK-16s) and Bcl6 (7D1) were used for intracellular staining using the eBioscience™ Foxp3/Transcription Factor Staining Buffer Set (eBioscience). Peripheral blood mononuclear cells (PBMCs) were collected as whole blood using sodium heparinized disposable microhaematocrit capillary tubes (Heamatokrit capillaries, Hirschmann Laborgerate GmbH&Co. KG, Eberstadt, Germany). Cells collected by the heparinized capillary tubes were placed into 96-well plates, washed, and stained with antibodies as described above.

For detecting RBD binding B cels, after Fc block with anti-CD16/32 Ab, cells were incubated with anti-CD4 (RM4-5), B220 (RA3-6B2), GL7 (GL7), CD38 (90), IgD (11-26c.2a), IgM mAbs (AF6-78) from Biolegend or eBioscience, and biotinylated S1 RBD protein or biotinylated-Ca04 HA for control. After washing, cells were then stained with stresptavidine-PE from BD Biosciences (Sparks, MD, USA).

Intracellular cytokine staining was performed on day 10 after injection with DT and SARS-CoV2 S1. One million cells from the draining lymph nodes and spleens were stimulated for 6 h with or without 1 μg/mL PepTivator SARS-CoV-2 Prot_S1, a SARS-CoV-2 spike peptide pool (S1 peptide) in the presence of GolgiStop (BD Biosciences, Sparks, MD, USA) in a U-bottom plate at 37°C under 5% CO_2_ in RPMI 1640 with 10% heat-inactivated FCS, 100 units/mL penicillin, and 100 μg/mL streptomycin. Cells incubated with 50 ng/mL PMA and 1 μg/mL ionomycin for 6 h were used as positive controls. After incubation, the cells were incubated with Live/dead fixable aqua and anti-CD16/32 Ab. Following fixation and permeabilization with Cytofix/Cytoperm from the Fixation/Permeabilization Solution Kit (BD Biosciences), cells were stained with antibodies specific to CD3 (17A2), CD45 (30-F11), CD4 (RM4-5), CD8a (53–6.7), and IFN-γ (XMG1.2) (Biolegend). Isotype control (RatIgG1κ) for IFN-γ was from Biolegend.

Data were acquired with Canto II flow cytometers (BD Bioscience) and data analysis was performed using FlowJo software (FlowJo, Ashland, OR, USA).

### Generation of Alexa Fluor 647-labeled SARS-CoV-2 S1 (S1^AF647^)protein and the analysis of S1^AF64^ -captured DCs

For the analysis of S1 protein uptake by DCs, SARS-CoV-2 whole S1 protein was conjugated to Alexa Fluor 647 using an Alexa Fluor™ 647 Microscale Protein Labeling Kit (Thermo Fisher Scientific), according to the manufacturer’s instructions. WT or *Foxp3*^DTR^ mice were i.p. injected with DT at 50 μg/kg on day 0, and immediately after DT injection, 5 μg of Alexa Fluor 647-labeled S1 *(S1*^*AF647*^*)* protein in 60 μl PBS were s.c. injected into the two fore footpads. On Day 2, DCs in axillarly lymph nodes as draining and inguinal lymph nodes as distal were analyzed by flow cytometry as described above.

### Treg-cell depletion by injecting anti-CD25 mAb

Anti-CD25 mAb (PC61) was purified from ascites, kindly provided by Dr. Osamu Taguchi, using HiTrap Protein G HP Columns from Cytiva (Marlborough, MA, USA), according to the manufacturer’s instructions. The endotoxin level of the purified antibody was < 0.0002 EU/μg, as determined using ToxinSensor™ Chromogenic LAL Endotoxin Assay Kit (Genscript, Piscataway, NJ, USA). WT mice were s.c. injected with 90 μg of anti-CD25 mAb and 10 μg of S1 protein in 120 μL PBS into all of the four footpads.

### Enzyme-linked Immunospot (ELISpot) assay

ELISpot was performed using the mouse interferon (IFN)-gamma ELISpot Kit (R&D Systems Inc., Minneapolis, USA or Abcam Inc., Cambridge, USA) according to the manufacture’s instructions.

Briefly, cells from the spleen or draining lymph nodes were isolated as described above and resuspended in RPMI 1640 with 10% FCS, 100 units/mL penicillin, and 100 μg/mL streptomycin at a concentration of 1 × 10^6^ cells/100μl/well. Subsequently, the cells were incubated with or without S1 peptide(PepTivator SARS-CoV-2 Prot_S1),at a final concentration of 1μg/mL in 96-well ELISpot plates at 37°C. Thirty-six hours later, the cells and supernatants were removed, and the ELISpot membranes were stained for IFN-γ. The plates were photographed by a digital camera with a dissecting microscope (Leica MZ10F, Leica Microsystem, Wetzlar, Germany) and numbers of spotos were quantified by counting manually.

### Enzyme-linked immunosorbent assay (ELISA) for SARS-CoV-2 S1-specific IgM and IgG

SARS-CoV-2 whole S1 protein or RBD protein in PBS (0.1 μg/50 μL/well) were coated onto 96 well plates overnight at 4°C. After being blocked with 2% non-fat milk for 1 h at room temperature, the plates were incubated with serum samples serially diluted in PBS for 1 h at room temperature. After washing with TBS plus 0.05% Tween-20 (TBS-T) (Nacalai Tesque, Kyoto, Japan) three times, the plates were incubated with an HRP-conjugated anti-mouse IgM (goat anti-mouse IgM μ chain from Abcam, Cambridge, UK) or IgG secondary antibody (MP Biomedicals, Santa Ana, CA, USA). Enzyme activity was detected by incubation with 100 μL of OPD substrate for 15 min at room temperature. The reaction was stopped by the addition of 100 μL of H_2_SO_4_. The optical density (OD) at 490 nm was measured using a microplate reader (SpectraMax340 PC384, Molecular Devices, LLC., San Jose, CA, USA).

### ELISA for total IgG and anti-dsDNA IgG

Total IgG and IgG against dsDNA levels were assessed by ELISA using the IgG (Total) Mouse Uncoated ELISA Kit (Thermo Fisher Scientific) and a mouse anti-dsDNA ELISA kit (Shibayagi, Gunma, Japan), respectively, according to the manufacturer’s instructions. Serum samples were diluted 1:100,000 for total IgG and 1:101 for IgG against dsDNA as recommended by the manufacturer. Enzyme activity was detected by incubation with 100 μL of 3, 3’, 5, 5’-tetramethylbenzidine (TMB) substrate for 15 min at room temperature. The reaction was stopped by the addition of 100μL of H_2_SO_4_. The OD at 450 nm was measured using a microplate reader SpectraMax340 PC384(Molecular Devices).

### SARS-CoV-2 plaque reduction neutralization tests (PRNT)

Fifty microliters of virus (about 100 plaques per well in a 6 well-plate) was incubated with 50 μL of three-fold serial dilutions of serum for 1 h at room temperature (15–25°C), and 100 μl of the mixture was added to confluent VeroE6/TMPRSS2 cells in 6-well plates, and incubated for 1 h at 37°C. After removing the mixture of virus and serum, the cells were overlaid with agarose in media and further incubated for 2 days at 37°C. Plaques were counted and virus neutralization titers were determined as the reciprocal of the highest serum dilution that reduced plaque numbers below 50% that of the control plates.

### Statistical analysis

GraphPad Prism 8 software was used to perform all statistical analysis. No statistical methods were used to predetermine sample size. A *P* value less than 0.05 was considered as statistically significant.

## Supporting information

S1 Fig Flow cytometry gating strategy. (A) Gating strategy for Figs 1B, 6B and S2. Foxp3^+^ CD4^+^ T cells are pre-gated on size, singlets, dead cell stain^−^, and CD45^+^. (B) Gating strategy for Figs 2B, S3A and S10. Foxp3^+^ or Foxp3^-^ CD4^+^ T cells are pre-gated on size, singlets, dead cell stain^−^, CD3^+^, CD4^+^, and separated by Foxp3 expression. (C) Gating strategy for Figs 2C and S3B. CD4^−^ B220^+^ B cells are pre-gated on size, singlets, and dead cell stain^−^. (D) Gating strategy for Fig 3B and 3C. MHCII^high^ CD11c^int^ migratory DCs, MHCII^int^ CD11c^high^ resident DCs and MHCII^+^ CD11c^-^ non-DCs are pre-gated on size, singlets, dead cell stain^−^, CD45^+^ and separated by MHCII and CD11c expression. (E) Gating strategy for Figs 5C, S8 and S9. CD3^+^ T or CD3^-^ cells pre-gated on size, singlets, dead cell stain^−^, and CD45^+^ and separated by CD3 expression.
(PDF)

S2 Fig Treg cells are recovered in draining lymph nodes and spleen on day 10. As in Fig 1B, but WT and *Foxp3*^DTR^ mice were injected with DT and S1 on day 0. On day 10, draining lymph nodes (axillary and popliteal) and spleen were analyzed. Representative graphs from two independent experiments (n = 3/group). Data were analyzed using unpaired Student’s t-test.
(PDF)

S3 Fig Transient Treg-cell depletion induces the formation of follicular helper T cells and GC B cells in spleen from S1-injected mice. (A) As in Fig 2A and 2B, but data from the spleen are shown. Representative gating strategy used to identify Tfr and Tfh cells (S1B Fig, gating strategy). Representative FACS plots of two independent experiments are shown. Representative graphs of two independent experiments are shown as the mean ± SEM (n = 3/group). Data were analyzed using unpaired Student’s t-test. (B) As in Fig 2C, but data from spleen are shown. Representative FACS plots of GL7^+^ CD38^-^germinal center (GC) B cells gated on live B220^+^ CD4^-^ cells (S1C Fig, gating strategy). IgM^-^ IgD^-^ class-switched GC B cells were gated on GL7^+^ CD38^-^GC B cells. RBD-binding class-switched GC B cells were gated on IgM^-^ IgD^-^ class-switched GC B cells. Influenza virus HA protein was used as the negative control for RBD-binding. Representative FACS plots of two independent experiments are shown. Representative frequencies of GC B, class-switched GC B, and RBD-binding GC B cells from two independent experiments are plotted as the mean ± SEM (n = 3/group). Data were analyzed using unpaired Student’s t-test.
(PDF)

S4 Fig Transient Treg-cell depletion induces small numbers of HA binding B cells non-specifically. As in Fig 2C, but HA-binding class-switched GC B cells were gated on IgM^-^ IgD^-^ class-switched GC B cells. HA protein was used as a negative control for RBD-binding. Representative frequencies of HA-binding GC B cells from two independent experiments are plotted as the mean ± SEM (n = 3/group). Data were analyzed using unpaired Student’s t-test.
(PDF)

S5 Fig Transient Treg-cell depletion induces maturation of DC subsets in SARS-CoV-2 S1-injected mice. (A) Representative FACS plots to identify CD11b^+^ and CD8^+^ subsets in migratory or resident DCs, as gated in Fig 3B, are shown from two independent experiments (top). The frequencies of CD11b^+^ and CD8^+^ DC subsets in resident or migratory DCs from draining lymph nodes 2 days after DT+S1-injection are representative data from two independent experiments (bottom). Data represent the mean ± SEM (DT+S1-injected WT mice, n = 4; DT+S1-injected *Foxp3*^DTR^ mice, n = 3). Data were analyzed using unpaired Student’s t-test. (B) As in (A), but CD80 and CD86 expression on CD11b^+^ or CD8^+^ DC subsets are shown. In histograms, cells were gated on CD11b^+^ migratory DCs, CD8^+^ migratory DCs, CD11b^+^ resident DCs or CD8^+^ resident DCs. The graphic data represented summaries from two independent experiments and represented as the mean ± SEM of the MFI (DT+S1-injected WT mice, n = 4; DT+S1-injected *Foxp3*^DTR^ mice, n = 3). Data were analyzed using unpaired Student’s t-test.
(PDF)

S6 Fig Treg-reduction induced by anti-CD25 monoclonal antibody does not induce SARS-CoV-2 S1-specific antibodies and DC maturation in mice. (A) A schematic diagram showing the experimental workflow. WT mice were injected s.c. into four footpads with anti-CD25 monoclonal antibody (mAb) (90 μg for experiment 1 and 250 μg for experiment 2) or control IgG, and 10 μg S1 protein on day 0. PBMCs on day 2, draining lymph nodes (axillary and popliteal), spleens and sera on day 10 were analyzed for Treg cells, DC maturation and antibodies. (B) PBMCs, draining lymph nodes and spleen from mice injected s.c. with S1 and 90 μg anti-CD25 mAb were analyzed for Foxp3 expression gated on live CD3^+^CD4^+^ cells. Data from experiment 1 with 90 μg anti-CD25 mAb is shown (PBMC, n = 4; draining lymph nodes and spleens, n = 3). Data present the mean ± SEM. Data were analyzed using unpaired Student’s t-test. (C) Representative histograms of CD80 and CD86 expression in resident DCs, migratory DCs, and MHCII^+^ CD11c^-^ non-DCs from draining lymph nodes of S1-injected WT mice treated with control IgG or anti-CD25 mAb (90 μg). The results of experiment 1 are shown (90 μg for experiment 1 and 250 μg for experiment 2; n = 1/group/experiment). In histograms, cells were gated on migratory DCs, resident DCs, or MHCII^+^ CD11c^-^ non-DCs as shown in Figs 3 and S1D. (D) Levels of S1 or RBD-specific IgG from the serum of S1-injected WT mice treated with control IgG or anti-CD25 mAb (90 μg) were determined by ELISA. Viral antibody endpoint titers against the whole S1 and RBD were expressed as the reciprocal of the highest dilution with an optical density at 490 nm (OD490) cutoff value > 0.1. n = 3 in a single experiment. Data represent the mean ± SEM. Data were analyzed using an unpaired Student’s t-test. The horizontal broken lines indicate the detection limits.
(PDF)

S7 Fig S1-captured DC subsets undergo maturation in transient Treg cell-depleted mice. (A) Representative FACS plots to identify CD11b^+^ and CD8^+^ subsets in S1^AF647^-captured migratory or resident DCs as gated in Fig 4D. Representative plots of two independent experiments are shown. (B) S1^AF647+^cell frequencies in the CD11b^+^ and CD8^+^ subsets in migratory or resident DCs in draining lymph nodes on day 2 after DT+S1^AF647^-injection. Representative of two independent experiments. The data are presented as the mean ± SEM (n = 3/group). Data were analyzed using two-way ANOVA with Tukey’s multiple comparisons test. (C) Representative overlaid contour plots of CD80 and CD86 expression gated on CD11b^+^ or CD8^+^ DC subsets within S1^AF647+^ resident or S1^AF647+^ migratory DC subsets from draining lymph nodes of DT+S1^AF647^-injected mice are shown (WT, green; *Foxp3*^DTR^, red-purple). Representative of two independent experiments for FACS plots and graphics are shown. The graphic data represents as the mean ± SEM of the MFI (n = 3/ group). Data were analyzed using an unpaired Student’s t-test.
(PDF)

S8 Fig SARS-CoV-2 antigen-specific IFN-γ producing CD4^+^ and CD8^+^ T cells are successfully induced in spleens from transient Treg cell-depleted mice. As in Fig 5C, but IFN-γ-secreting cells from spleens are shown in CD4^+^ (left) and CD8^+^ T (right) cells. Representative plots from two independent experiments are shown, gated on live CD45^+^, CD3^+^ cells (S1E Fig., gating strategy). The frequencies of positive cell populations are represented as the mean ± SEM(n = 3/group). Representative from two independent experiments. Data were analyzed using two-way ANOVA with Tukey’s multiple comparisons test.
(PDF)

S9 Fig IFN-γ producing CD3^-^ cells are induced in transient Treg cell-depleted mice. As in Fig 5C, but cells from draining lymph nodes (left) and spleens (right) are shown for IFN-γ expression in CD3^-^ cells. Representative plots from two independent experiments that were pre-gated on size, singlets, dead cell stain^−^,CD45^+^, and CD3^-^ cells (S1E Fig, gating strategy). Representative of two independent experiments for FACS plots and graphics are shown. The mean frequencies of positive cell populations are represented ± SEM (n = 3/group). Data were analyzed using two-way ANOVA with Tukey’s multiple comparisons test.
(PDF)

S10 Fig Treg cells are recovered in draining lymph nodes on day 10 with poly IC. As in Fig 6A, but WT and *Foxp3*^DTR^ mice were injected with DT and S1 on day 0 with or without poly IC. On day 10, draining lymph nodes (axillary and popliteal) were analyzed. Representative graphs from a single experiment (n = 3/group), gated on CD3^+^ CD4^+^ cells (S1B Fig, gating strategy). Data were analyzed using two-way ANOVA with Tukey’s multiple comparisons test.
(PDF)
