# Supplementary material for: Foxp3+ CD4+ regulatory T cells control dendritic cells in inducing antigen-specific immunity to emerging SARS-CoV-2 antigens
Source: PLoS Pathog. 2021 Dec 9;17(12):e1010085. doi: 10.1371/journal.ppat.1010085 (PMC8659413; doi:10.1371/journal.ppat.1010085)
Supplement: S1 Fig — (A) Gating strategy for Figs 1B, 6B and S2. Foxp3+ CD4+ T cells are pre-gated on size, singlets, dead cell stain−, and CD45+. (B) Gating strategy for Figs 2B, S3A and S10. Foxp3+ or Foxp3- CD4+ T cells are pre-gated on size, singlets, dead cell stain−, CD3+, CD4+, and separated by Foxp3 expression. (C) Gating strategy for Figs 2C and S3B. CD4− B220+ B cells are pre-gated on size, singlets, and dead cell stain−. (D) Gating strategy for Fig 3B and 3C. MHCIIhigh CD11cint migratory DCs, MHCIIint CD11chigh resident DCs and MHCII+ CD11c- non-DCs are pre-gated on size, singlets, dead cell stain−, CD45+ and separated by MHCII and CD11c expression. (E) Gating strategy for Figs 5C, S8 and S9. CD3+ T or CD3- cells pre-gated on size, singlets, dead cell stain−, and CD45+ and separated by CD3 expression. (PDF) [file ppat.1010085.s001.pdf]

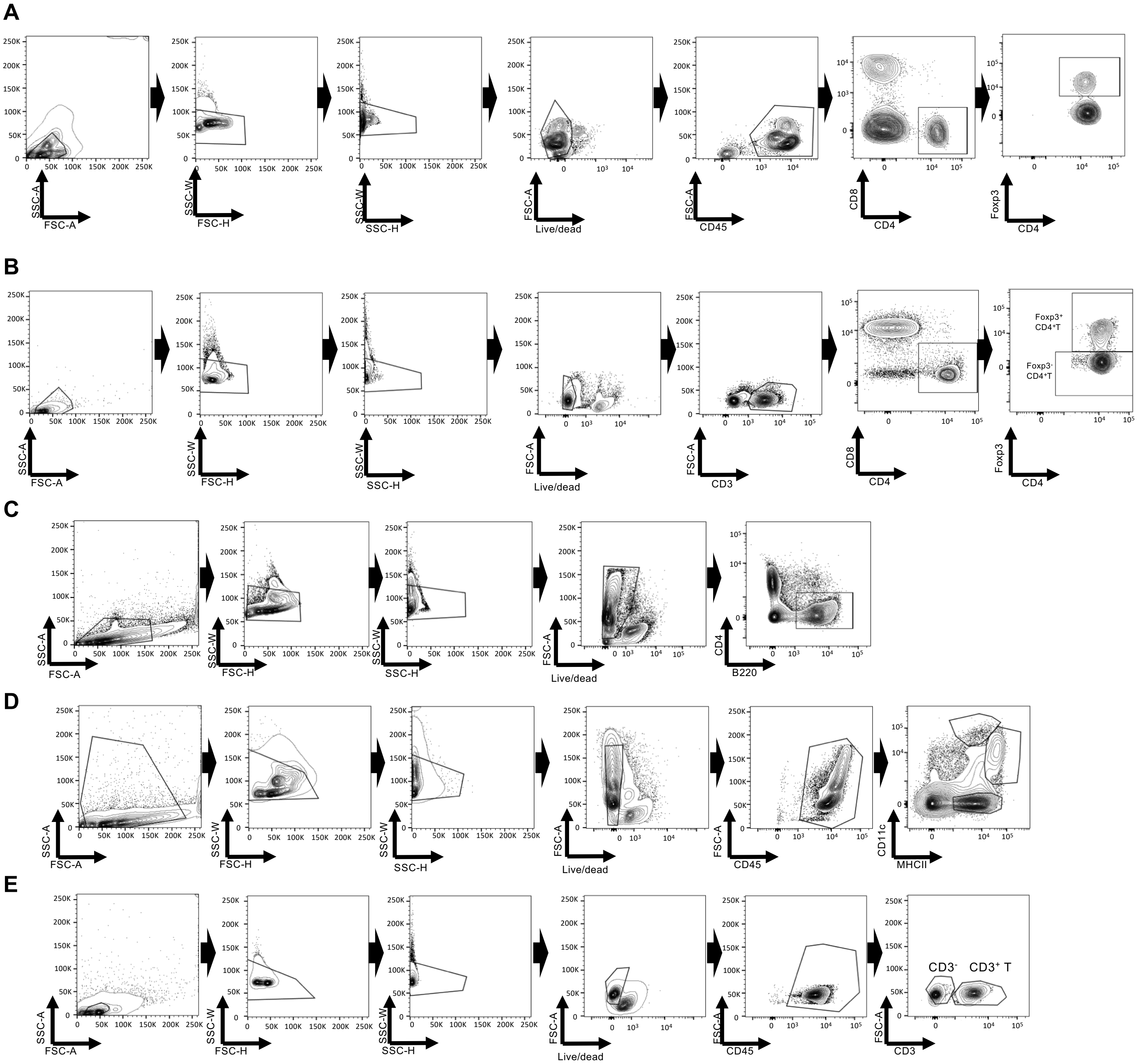

S1 Fig. Flow cytometry gating strategy

(A) Gating strategy for Figs 1B, 6B and S2. Foxp3^{+} CD4^{+} T cells are pre-gated on size, singlets, dead cell stain^{-}, and CD45^{+}.

(B) Gating strategy for Figs 2B, S3A and S10. Foxp3^{+} or Foxp3^{-} CD4^{+} T cells are pre-gated on size, singlets, dead cell stain^{-}, CD3^{+}, CD4^{+}, and separated by Foxp3 expression.

(C) Gating strategy for Figs 2C and S3B. CD4^{-} B220^{+} B cells are pre-gated on size, singlets, and dead cell stain^{-}.

(D) Gating strategy for Figs 3B and 3C. MHCII^{high} CD11c^{int} migratory DCs, MHCII^{int} CD11c^{high} resident DCs and MHCII^{+} CD11c^{-} non-DCs are pre-gated on size, singlets, dead cell stain^{-}, CD45^{+} and separated by MHCII and CD11c expression.

(E) Gating strategy for Figs 5C, S8 and S9. CD3^{+} T or CD3^{-} cells pre-gated on size, singlets, dead cell stain^{-}, and CD45^{+} and separated by CD3 expression.
