# Supplementary material for: Foxp3+ CD4+ regulatory T cells control dendritic cells in inducing antigen-specific immunity to emerging SARS-CoV-2 antigens
Source: PLoS Pathog. 2021 Dec 9;17(12):e1010085. doi: 10.1371/journal.ppat.1010085 (PMC8659413; doi:10.1371/journal.ppat.1010085)
Supplement: S2 Fig — As in Fig 1B, but WT and Foxp3DTR mice were injected with DT and S1 on day 0. On day 10, draining lymph nodes (axillary and popliteal) and spleen were analyzed. Representative graphs from two independent experiments (n = 3/group). Data were analyzed using unpaired Student’s t-test. (PDF) [file ppat.1010085.s002.pdf]

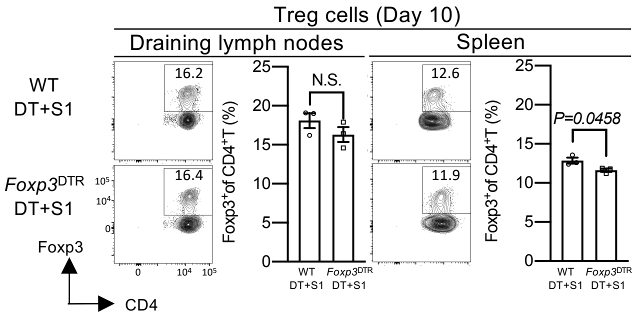

S2 Fig. Treg cells are recovered in draining lymph nodes and spleen on day 10.

As in Fig 1B, but WT and *Foxp3*^{DTR} mice were injected with DT and S1 on day 0. On day 10, draining lymph nodes (axillary and popliteal) and spleen were analyzed. Representative graphs from two independent experiments (n = 3/group). Data were analyzed using unpaired Student's t-test.
