# Supplementary material for: Foxp3+ CD4+ regulatory T cells control dendritic cells in inducing antigen-specific immunity to emerging SARS-CoV-2 antigens
Source: PLoS Pathog. 2021 Dec 9;17(12):e1010085. doi: 10.1371/journal.ppat.1010085 (PMC8659413; doi:10.1371/journal.ppat.1010085)
Supplement: S3 Fig — (A) As in Fig 2A and 2B, but data from the spleen are shown. Representative gating strategy used to identify Tfr and Tfh cells (S1B Fig, gating strategy). Representative FACS plots of two independent experiments are shown. Representative graphs of two independent experiments are shown as the mean ± SEM (n = 3/group). Data were analyzed using unpaired Student’s t-test. (B) As in Fig 2C, but data from spleen are shown. Representative FACS plots of GL7+ CD38-germinal center (GC) B cells gated on live B220+ CD4- cells (S1C Fig, gating strategy). IgM- IgD- class-switched GC B cells were gated on GL7+ CD38-GC B cells. RBD-binding class-switched GC B cells were gated on IgM- IgD- class-switched GC B cells. Influenza virus HA protein was used as the negative control for RBD-binding. Representative FACS plots of two independent experiments are shown. Representative frequencies of GC B, class-switched GC B, and RBD-binding GC B cells from two independent experiments are plotted as the mean ± SEM (n = 3/group). Data were analyzed using unpaired Student’s t-test. (PDF) [file ppat.1010085.s003.pdf]

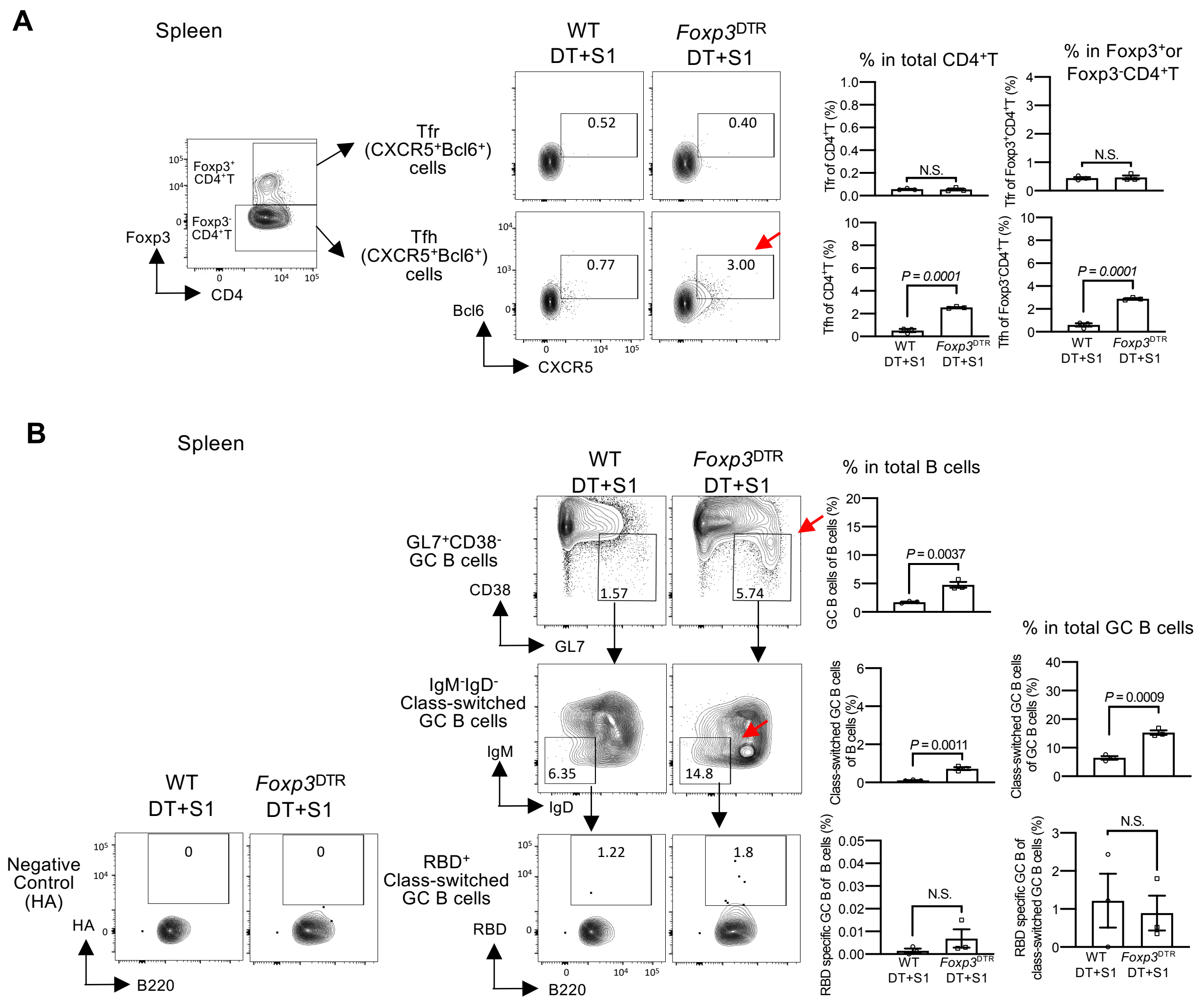

S3 Fig. Transient Treg depletion induces the formation of follicular helper T cells and GC B cells in spleen from S1-injected mice.

(A) As in Fig 2A, B, but data from the spleen are shown. Representative gating strategy used to identify Tfr and Tfh cells (S1B Fig, gating strategy). Representative FACS plots of two independent experiments are shown. Representative graphs of two independent experiments are shown as the mean ± SEM (n = 3/group). Data were analyzed using unpaired Student's t-test.

(B) As in Fig 2C, but data from spleen are shown. Representative FACS plots of GL7^{+} CD38^{-}germinal center (GC) B cells gated on live B220^{+} CD4^{-} cells (S1C Fig, gating strategy). IgM^{-} IgD^{-} class-switched GC B cells were gated on GL7^{+} CD38^{-}GC B cells. RBD-binding class-switched GC B cells were gated on IgM^{-} IgD^{-} class-switched GC B cells. Influenza virus HA protein was used as the negative control for RBD-binding. Representative FACS plots of two independent experiments are shown. Representative frequencies of GC B, class-switched GC B, and RBD-binding GC B cells from two independent experiments are plotted as the mean ± SEM (n = 3/group). Data were analyzed using unpaired Student's t-test.
