# Supplementary material for: Foxp3+ CD4+ regulatory T cells control dendritic cells in inducing antigen-specific immunity to emerging SARS-CoV-2 antigens
Source: PLoS Pathog. 2021 Dec 9;17(12):e1010085. doi: 10.1371/journal.ppat.1010085 (PMC8659413; doi:10.1371/journal.ppat.1010085)
Supplement: S4 Fig — As in Fig 2C, but HA-binding class-switched GC B cells were gated on IgM- IgD- class-switched GC B cells. HA protein was used as a negative control for RBD-binding. Representative frequencies of HA-binding GC B cells from two independent experiments are plotted as the mean ± SEM (n = 3/group). Data were analyzed using unpaired Student’s t-test. (PDF) [file ppat.1010085.s004.pdf]

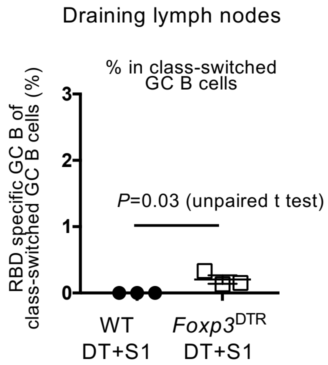

S4 Fig. Transient Treg-cell depletion induces small numbers of HA binding B cells non-specifically.
As in Fig 2C, but HA-binding class-switched GC B cells were gated on IgM^- IgD^- class-switched GC B cells. HA protein was used as a negative control for RBD-binding. Representative frequencies of HA-binding GC B cells from two independent experiments are plotted as the mean ± SEM (n = 3/group). Data were analyzed using unpaired Student's t-test.
