# Supplementary material for: Foxp3+ CD4+ regulatory T cells control dendritic cells in inducing antigen-specific immunity to emerging SARS-CoV-2 antigens
Source: PLoS Pathog. 2021 Dec 9;17(12):e1010085. doi: 10.1371/journal.ppat.1010085 (PMC8659413; doi:10.1371/journal.ppat.1010085)
Supplement: S5 Fig — (A) Representative FACS plots to identify CD11b+ and CD8+ subsets in migratory or resident DCs, as gated in Fig 3B, are shown from two independent experiments (top). The frequencies of CD11b+ and CD8+ DC subsets in resident or migratory DCs from draining lymph nodes 2 days after DT+S1-injection are representative data from two independent experiments (bottom). Data represent the mean ± SEM (DT+S1-injected WT mice, n = 4; DT+S1-injected Foxp3DTR mice, n = 3). Data were analyzed using unpaired Student’s t-test. (B) As in (A), but CD80 and CD86 expression on CD11b+ or CD8+ DC subsets are shown. In histograms, cells were gated on CD11b+ migratory DCs, CD8+ migratory DCs, CD11b+ resident DCs or CD8+ resident DCs. The graphic data represented summaries from two independent experiments and represented as the mean ± SEM of the MFI (DT+S1-injected WT mice, n = 4; DT+S1-injected Foxp3DTR mice, n = 3). Data were analyzed using unpaired Student’s t-test. (PDF) [file ppat.1010085.s005.pdf]

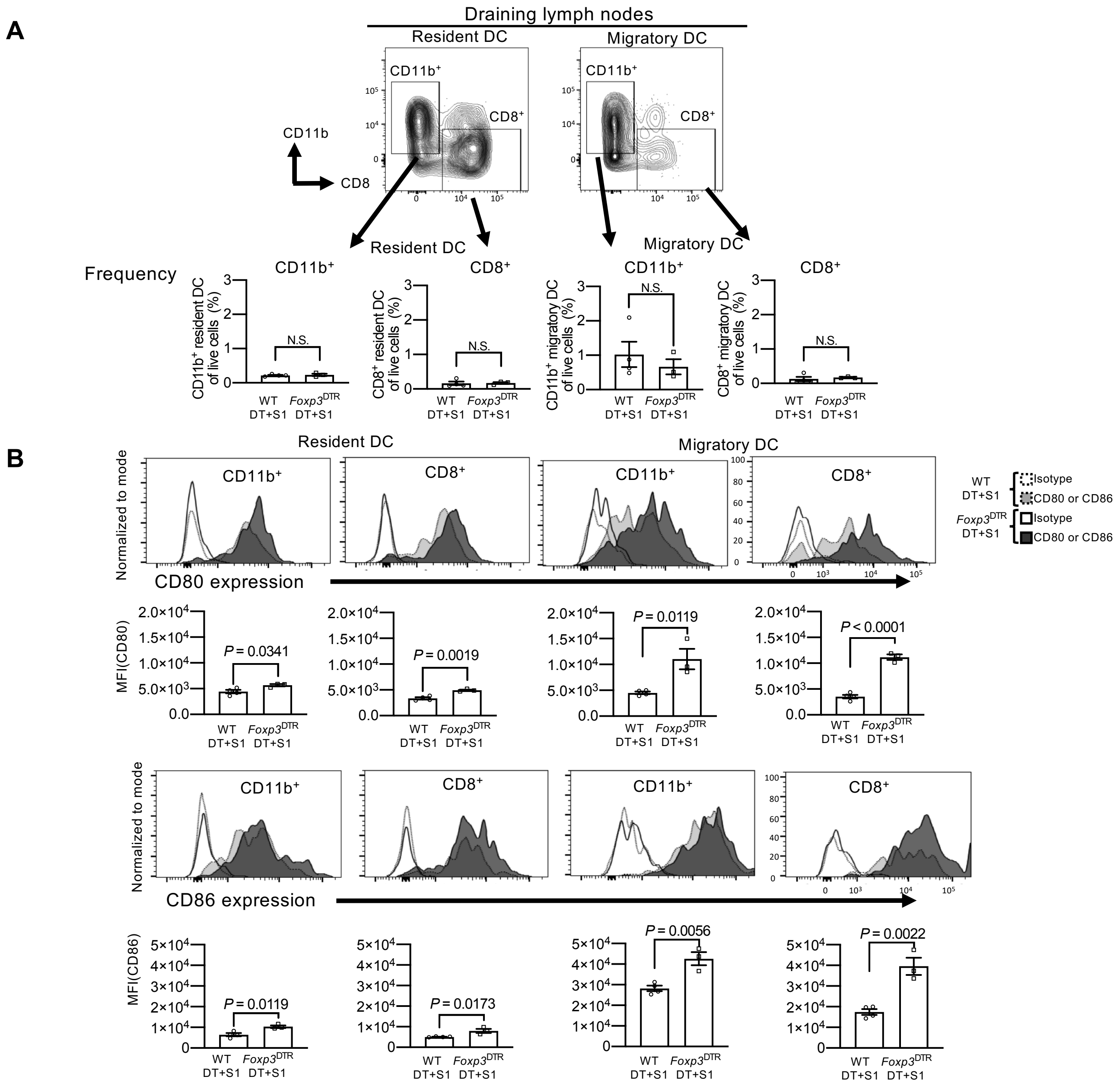

S5 Fig. Transient Treg-cell depletion induces maturation of DC subsets in SARS-CoV-2 S1-injected mice.

(A) Representative FACS plots to identify $CD11b^+$ and $CD8^+$ subsets in migratory or resident DCs, as gated in Fig 3B, are shown from two independent experiments (top). The frequencies of $CD11b^+$ and $CD8^+$ DC subsets in resident or migratory DCs from draining lymph nodes 2 days after DT+S1-injection are representative data from two independent experiments (bottom). Data represent the mean ± SEM (DT+S1-injected WT mice, n = 4; DT+S1-injected *Foxp3*^{DTR} mice, n = 3). Data were analyzed using unpaired Student's t-test.

(B) As in (A), but CD80 and CD86 expression on $CD11b^+$ or $CD8^+$ DC subsets are shown. In histograms, cells were gated on $CD11b^+$ migratory DCs, $CD8^+$ migratory DCs, $CD11b^+$ resident DCs or $CD8^+$ resident DCs. The graphic data represented summaries from two independent experiments and represented as the mean ± SEM of the MFI (DT+S1-injected WT mice, n = 4; DT+S1-injected *Foxp3*^{DTR} mice, n = 3). Data were analyzed using unpaired Student's t-test.
