# Supplementary material for: Foxp3+ CD4+ regulatory T cells control dendritic cells in inducing antigen-specific immunity to emerging SARS-CoV-2 antigens
Source: PLoS Pathog. 2021 Dec 9;17(12):e1010085. doi: 10.1371/journal.ppat.1010085 (PMC8659413; doi:10.1371/journal.ppat.1010085)
Supplement: S6 Fig — (A) A schematic diagram showing the experimental workflow. WT mice were injected s.c. into four footpads with anti-CD25 monoclonal antibody (mAb) (90 μg for experiment 1 and 250 μg for experiment 2) or control IgG, and 10 μg S1 protein on day 0. PBMCs on day 2, draining lymph nodes (axillary and popliteal), spleens and sera on day 10 were analyzed for Treg cells, DC maturation and antibodies. (B) PBMCs, draining lymph nodes and spleen from mice injected s.c. with S1 and 90 μg anti-CD25 mAb were analyzed for Foxp3 expression gated on live CD3+CD4+ cells. Data from experiment 1 with 90 μg anti-CD25 mAb is shown (PBMC, n = 4; draining lymph nodes and spleens, n = 3). Data present the mean ± SEM. Data were analyzed using unpaired Student’s t-test. (C) Representative histograms of CD80 and CD86 expression in resident DCs, migratory DCs, and MHCII+ CD11c- non-DCs from draining lymph nodes of S1-injected WT mice treated with control IgG or anti-CD25 mAb (90 μg). The results of experiment 1 are shown (90 μg for experiment 1 and 250 μg for experiment 2; n = 1/group/experiment). In histograms, cells were gated on migratory DCs, resident DCs, or MHCII+ CD11c- non-DCs as shown in Figs 3 and S1D. (D) Levels of S1 or RBD-specific IgG from the serum of S1-injected WT mice treated with control IgG or anti-CD25 mAb (90 μg) were determined by ELISA. Viral antibody endpoint titers against the whole S1 and RBD were expressed as the reciprocal of the highest dilution with an optical density at 490 nm (OD490) cutoff value > 0.1. n = 3 in a single experiment. Data represent the mean ± SEM. Data were analyzed using an unpaired Student’s t-test. The horizontal broken lines indicate the detection limits. (PDF) [file ppat.1010085.s006.pdf]

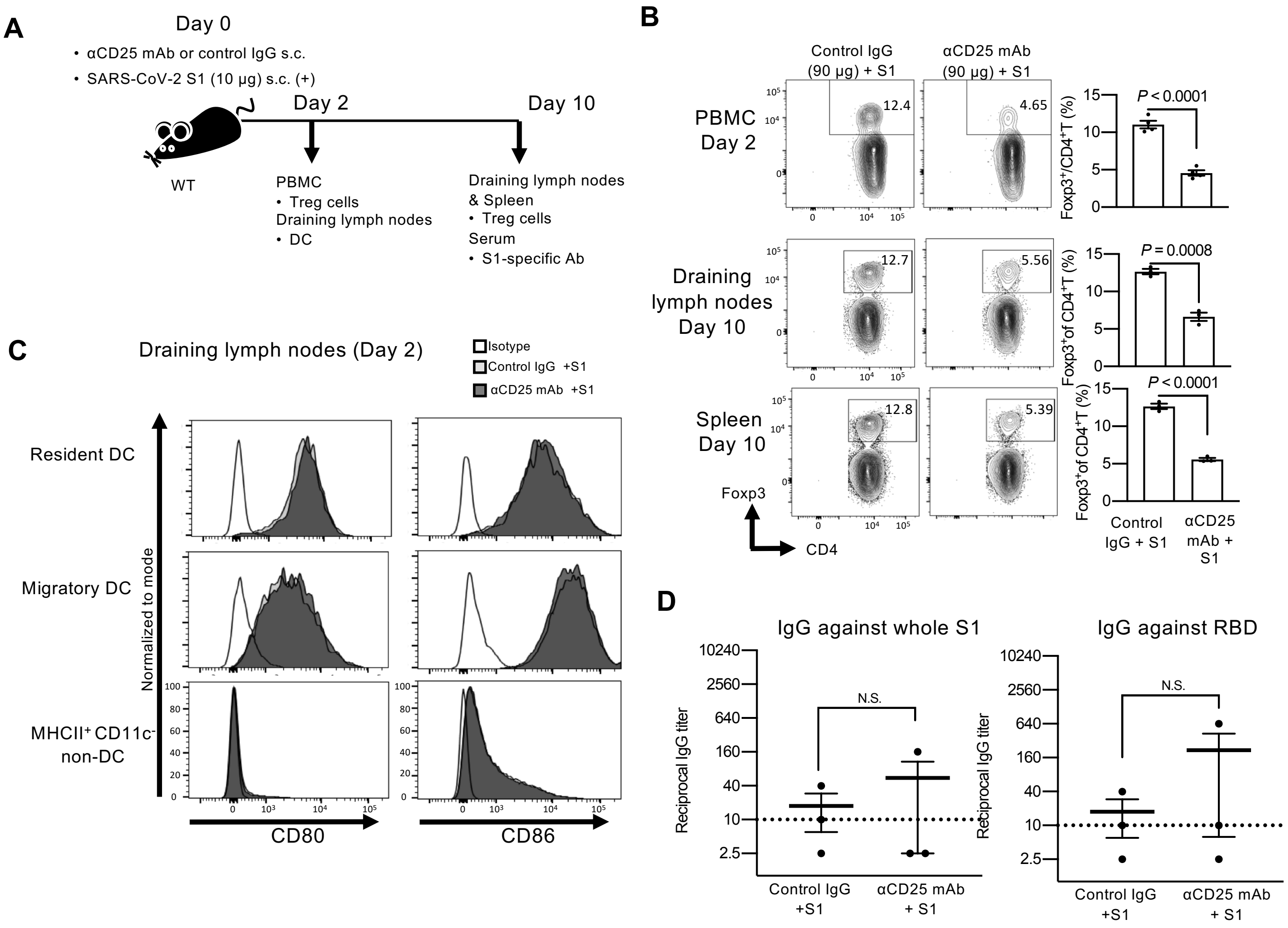

S6 Fig. Treg-reduction induced by anti-CD25 monoclonal antibody does not induce SARS-CoV-2 S1-specific antibodies and DC maturation in mice.

(A) A schematic diagram showing the experimental workflow. WT mice were injected s.c. into four footpads with anti-CD25 monoclonal antibody (mAb) (90 µg for experiment 1 and 250 µg for experiment 2) or control IgG, and 10 µg S1 protein on day 0. PBMCs on day 2, draining lymph nodes (axillary and popliteal), spleens and sera on day 10 were analyzed for Treg cells, DC maturation and antibodies.

(B) PBMCs, draining lymph nodes and spleen from mice injected s.c. with S1 and 90 µg anti-CD25 mAb were analyzed for Foxp3 expression gated on live $CD3^+CD4^+$ cells. Data from experiment 1 with 90 µg anti-CD25 mAb is shown (PBMC, n = 4; draining lymph nodes and spleens, n = 3). Data present the mean ± SEM. Data were analyzed using unpaired Student's t-test.

(C) Representative histograms of CD80 and CD86 expression in resident DCs, migratory DCs, and $MHCII^+$ $CD11c^-$ non-DCs from draining lymph nodes of S1-injected WT mice treated with control IgG or anti-CD25 mAb (90 µg). The results of experiment 1 are shown (90 µg for experiment 1 and 250 µg for experiment 2; n = 1/group/experiment). In histograms, cells were gated on migratory DCs, resident DCs, or $MHCII^+$ $CD11c^-$ non-DCs as shown in Figs 3 and S1D.

(D) Levels of S1 or RBD-specific IgG from the serum of S1-injected WT mice treated with control IgG or anti-CD25 mAb (90 µg) were determined by ELISA. Viral antibody endpoint titers against the whole S1 and RBD were expressed as the reciprocal of the highest dilution with an optical density at 490 nm (OD490) cutoff value > 0.1. n=3 in a single experiment. Data represent the mean ± SEM. Data were analyzed using an unpaired Student's t-test. The horizontal broken lines indicate the detection limits.
