# Supplementary material for: Foxp3+ CD4+ regulatory T cells control dendritic cells in inducing antigen-specific immunity to emerging SARS-CoV-2 antigens
Source: PLoS Pathog. 2021 Dec 9;17(12):e1010085. doi: 10.1371/journal.ppat.1010085 (PMC8659413; doi:10.1371/journal.ppat.1010085)
Supplement: S7 Fig — (A) Representative FACS plots to identify CD11b+ and CD8+ subsets in S1AF647-captured migratory or resident DCs as gated in Fig 4D. Representative plots of two independent experiments are shown. (B) S1AF647+cell frequencies in the CD11b+ and CD8+ subsets in migratory or resident DCs in draining lymph nodes on day 2 after DT+S1AF647-injection. Representative of two independent experiments. The data are presented as the mean ± SEM (n = 3/group). Data were analyzed using two-way ANOVA with Tukey’s multiple comparisons test. (C) Representative overlaid contour plots of CD80 and CD86 expression gated on CD11b+ or CD8+ DC subsets within S1AF647+ resident or S1AF647+ migratory DC subsets from draining lymph nodes of DT+S1AF647-injected mice are shown (WT, green; Foxp3DTR, red-purple). Representative of two independent experiments for FACS plots and graphics are shown. The graphic data represents as the mean ± SEM of the MFI (n = 3/ group). Data were analyzed using an unpaired Student’s t-test. (PDF) [file ppat.1010085.s007.pdf]

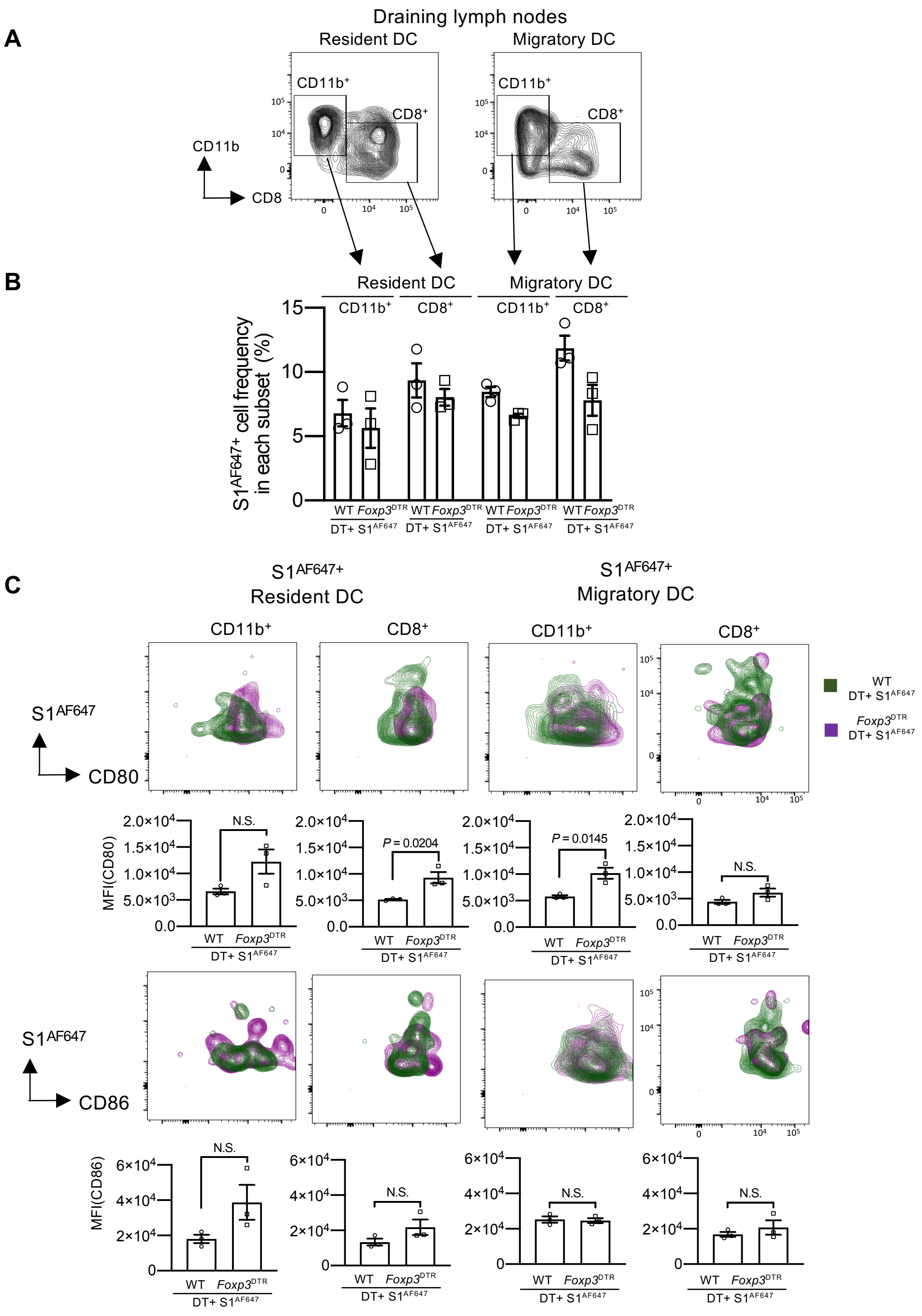

S7 Fig. S1-captured DC subsets undergo maturation in transient Treg cell-depleted mice.

(A) Representative FACS plots to identify CD11b$^+$ and CD8$^+$ subsets in S1^{AF647}-captured migratory or resident DCs as gated in Fig 4D. Representative plots of two independent experiments are shown.

(B) S1^{AF647+}cell frequencies in the CD11b$^+$ and CD8$^+$ subsets in migratory or resident DCs in draining lymph nodes on day 2 after DT+S1^{AF647}-injection. Representative of two independent experiments. The data are presented as the mean ± SEM (n = 3/group). Data were analyzed using two-way ANOVA with Tukey's multiple comparisons test.

(C) Representative overlaid contour plots of CD80 and CD86 expression gated on CD11b$^+$ or CD8$^+$ DC subsets within S1^{AF647+} resident or S1^{AF647+} migratory DC subsets from draining lymph nodes of DT+S1^{AF647}-injected mice are shown (WT, green; *Foxp3*^{DTR}, red-purple). Representative of two independent experiments for FACS plots and graphics are shown. The graphic data represents as the mean ± SEM of the MFI (n=3/ group). Data were analyzed using an unpaired Student's t-test.
