# Supplementary material for: Foxp3+ CD4+ regulatory T cells control dendritic cells in inducing antigen-specific immunity to emerging SARS-CoV-2 antigens
Source: PLoS Pathog. 2021 Dec 9;17(12):e1010085. doi: 10.1371/journal.ppat.1010085 (PMC8659413; doi:10.1371/journal.ppat.1010085)
Supplement: S8 Fig — As in Fig 5C, but IFN-γ-secreting cells from spleens are shown in CD4+ (left) and CD8+ T (right) cells. Representative plots from two independent experiments are shown, gated on live CD45+, CD3+ cells (S1E Fig., gating strategy). The frequencies of positive cell populations are represented as the mean ± SEM(n = 3/group). Representative from two independent experiments. Data were analyzed using two-way ANOVA with Tukey’s multiple comparisons test. (PDF) [file ppat.1010085.s008.pdf]

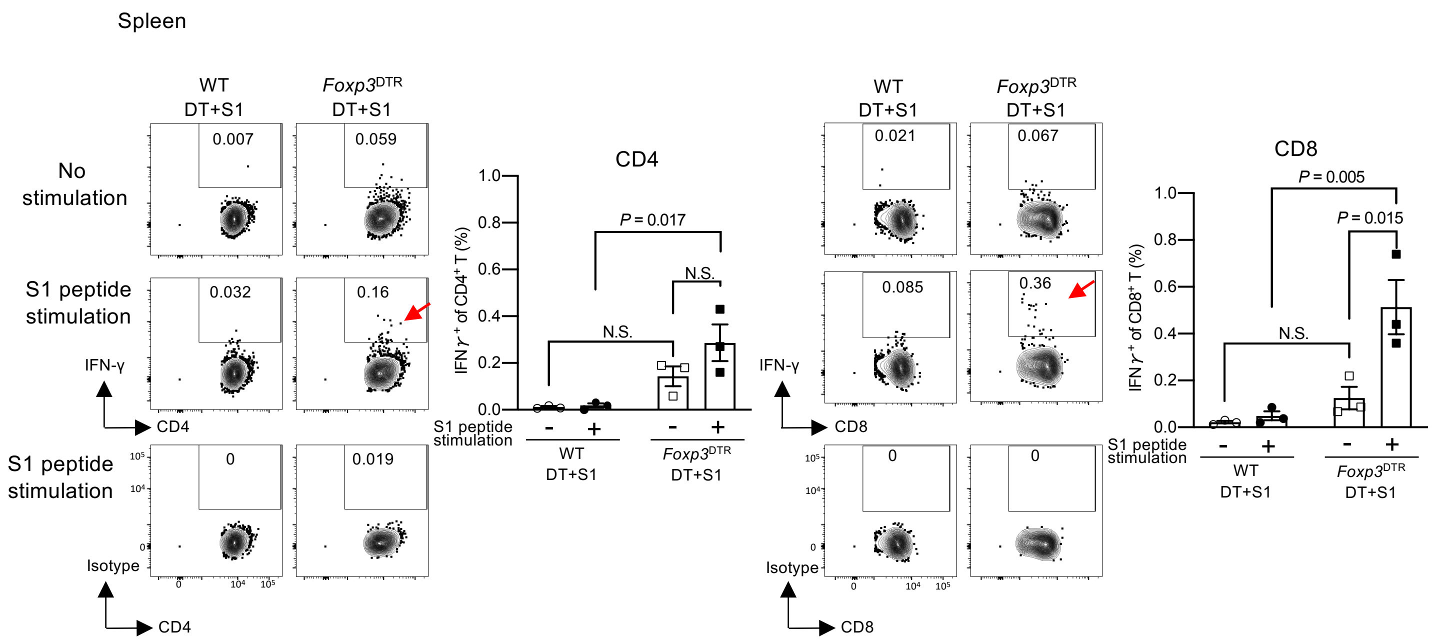

S8 Fig. SARS-CoV-2 antigen-specific IFN-γ producing CD4$^+$ and CD8$^+$ T cells are successfully induced in spleens from transient Treg cell-depleted mice.

As in Fig 5C, but IFN-γ-secreting cells from spleens are shown in CD4$^+$ (left) and CD8$^+$ T (right) cells. Representative plots from two independent experiments are shown, gated on live CD45$^+$, CD3$^+$ cells (S1E Fig., gating strategy). The frequencies of positive cell populations are represented as the mean ± SEM(n = 3/group). Representative from two independent experiments. Data were analyzed using two-way ANOVA with Tukey's multiple comparisons test.
