# Supplementary material for: Foxp3+ CD4+ regulatory T cells control dendritic cells in inducing antigen-specific immunity to emerging SARS-CoV-2 antigens
Source: PLoS Pathog. 2021 Dec 9;17(12):e1010085. doi: 10.1371/journal.ppat.1010085 (PMC8659413; doi:10.1371/journal.ppat.1010085)
Supplement: S9 Fig — As in Fig 5C, but cells from draining lymph nodes (left) and spleens (right) are shown for IFN-γ expression in CD3- cells. Representative plots from two independent experiments that were pre-gated on size, singlets, dead cell stain−,CD45+, and CD3- cells (S1E Fig, gating strategy). Representative of two independent experiments for FACS plots and graphics are shown. The mean frequencies of positive cell populations are represented ± SEM (n = 3/group). Data were analyzed using two-way ANOVA with Tukey’s multiple comparisons test. (PDF) [file ppat.1010085.s009.pdf]

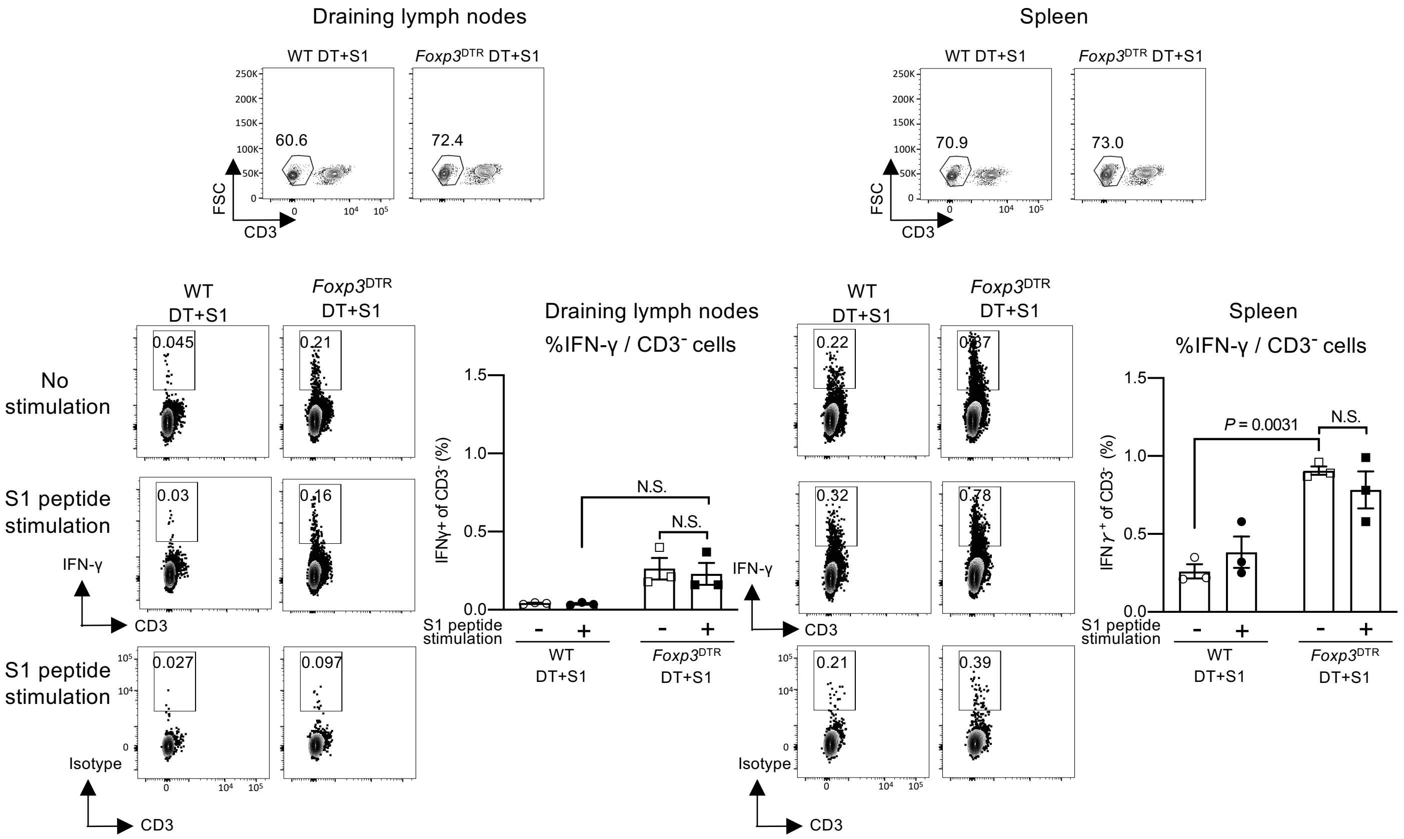

S9 Fig. IFN-γ producing CD3⁻ cells are induced in transient Treg cell-depleted mice.
As in Fig 5C, but cells from draining lymph nodes (left) and spleens (right) are shown for IFN-γ expression in CD3⁻ cells. Representative plots from two independent experiments that were pre-gated on size, singlets, dead cell stain⁻,CD45⁺, and CD3⁻ cells (S1E Fig, gating strategy). Representative of two independent experiments for FACS plots and graphics are shown. The mean frequencies of positive cell populations are represented ± SEM (n = 3/group). Data were analyzed using two-way ANOVA with Tukey's multiple comparisons test.
