# Supplementary material for: Foxp3+ CD4+ regulatory T cells control dendritic cells in inducing antigen-specific immunity to emerging SARS-CoV-2 antigens
Source: PLoS Pathog. 2021 Dec 9;17(12):e1010085. doi: 10.1371/journal.ppat.1010085 (PMC8659413; doi:10.1371/journal.ppat.1010085)
Supplement: S10 Fig — As in Fig 6A, but WT and Foxp3DTR mice were injected with DT and S1 on day 0 with or without poly IC. On day 10, draining lymph nodes (axillary and popliteal) were analyzed. Representative graphs from a single experiment (n = 3/group), gated on CD3+ CD4+ cells (S1B Fig, gating strategy). Data were analyzed using two-way ANOVA with Tukey’s multiple comparisons test. (PDF) [file ppat.1010085.s010.pdf]

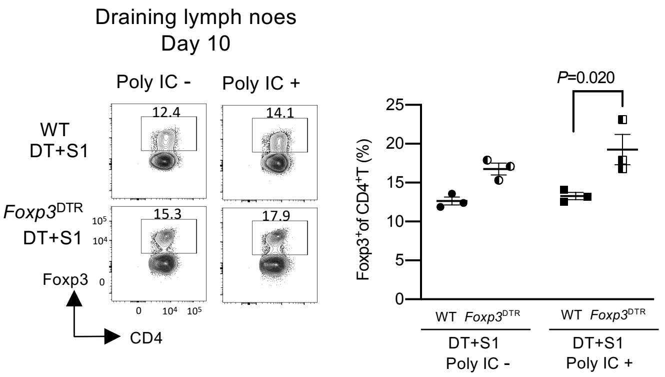

S10 Fig. Treg cells are recovered in draining lymph nodes on day 10 with poly IC.

As in Fig 6A, but WT and *Foxp3*^{DTR} mice were injected with DT and S1 on day 0 with or without poly IC. On day 10, draining lymph nodes (axillary and popliteal) were analyzed. Representative graphs from a single experiment (n = 3/group), gated on $CD3^{+}$ $CD4^{+}$ cells (S1B Fig, gating strategy). Data were analyzed using two-way ANOVA with Tukey's multiple comparisons test.
